# Novel Serum and Urinary Metabolites Associated with Diabetic Retinopathy in Three Asian Cohorts

**DOI:** 10.3390/metabo11090614

**Published:** 2021-09-09

**Authors:** Debra Q. Y. Quek, Feng He, Rehena Sultana, Riswana Banu, Miao Li Chee, Simon Nusinovici, Sahil Thakur, Chaoxu Qian, Ching-Yu Cheng, Tien Y. Wong, Charumathi Sabanayagam

**Affiliations:** 1 Singapore Eye Research Institute, Singapore National Eye Centre, Singapore 168751, Singapore; debra.quek@mohh.com.sg (D.Q.Y.Q.); e0215973@u.nus.edu (F.H.); riswana.banu.mohd.abdul@seri.com.sg (R.B.); chee.miao.li@seri.com.sg (M.L.C.); simon.nusinovici@seri.com.sg (S.N.); thakur.sahil@seri.com.sg (S.T.); qian.chaoxu@seri.com.sg (C.Q.); chingyu.cheng@duke-nus.edu.sg (C.-Y.C.); wong.tien.yin@singhealth.com.sg (T.Y.W.); 2 Centre for Quantitative Medicine, Duke-NUS Medical School, Singapore 169857, Singapore; rehena.sultana@duke-nus.edu.sg; 3 Ophthalmology and Visual Sciences Academic Clinical Program, Duke-NUS Medical School, Singapore 169857, Singapore

**Keywords:** serum/plasma metabolites, urinary metabolites, diabetic retinopathy, nuclear magnetic resonance spectroscopy, cross-sectional study

## Abstract

Diabetic retinopathy (DR) is a microvascular complication of diabetes mellitus, a metabolic disorder, but understanding of its pathophysiology remains incomplete. Meta-analysis of three population-based cross-sectional studies (2004–11) representing three major Asian ethnic groups (aged 40–80 years: Chinese, 592; Malays, 1052; Indians, 1320) was performed. A panel of 228 serum/plasma metabolites and 54 urinary metabolites were quantified using nuclear magnetic resonance (NMR) spectroscopy. Main outcomes were defined as any DR, moderate/above DR, and vision-threatening DR assessed from retinal photographs. The relationship between metabolites and DR outcomes was assessed using multivariate logistic regression models, and metabolites significant after Bonferroni correction were meta-analyzed. Among serum/plasma metabolites, lower levels of tyrosine and cholesterol esters to total lipids ratio in IDL and higher levels of creatinine were positively associated with all three outcomes of DR (all *p* < 0.005). Among urinary metabolites, lower levels of citrate, ethanolamine, formate, and hypoxanthine were positively associated with all three DR outcomes (all *p* < 0.005). Higher levels of serum/plasma 3-hydroxybutyrate and lower levels of urinary 3-hydroxyisobutyrate were associated with VTDR. Comprehensive metabolic profiling in three large Asian cohorts with DR demonstrated alterations in serum/plasma and urinary metabolites mostly related to amino acids, lipoprotein subclasses, kidney function, and glycolysis.

## 1. Introduction

In 2019, an estimated 463 million adults (20–79 years old) globally had diabetes, and this is expected to increase to 700 million by 2045 [1]. Diabetic retinopathy (DR) is one of the most common microvascular complications of diabetes, and is the leading cause of preventable blindness in working-age adults globally [2,3]. With the rising prevalence of diabetes and the increasing life expectancy of people with diabetes, the number of people with DR is also expected to rise.

Diabetes mellitus is a metabolic disorder associated with insulin resistance, altered lipid metabolism, subclinical inflammation, and oxidative stress [4,5,6]. Metabolic alterations have also been reported amongst the complications of diabetes, along with microvascular complications such as DR [7,8,9,10,11,12]. Sustained hyperglycemia, hypertension, and dyslipidemia are key risk factors for the development of DR [2,3,13]. However, these risk factors do not fully account for DR risk, suggesting that additional risk factors play a role in DR pathogenesis [13].

Two main techniques of metabolomic profiling are currently used—mass-spectrometry (MS) or nuclear magnetic resonance (NMR). These techniques provide complementary insights, due to their respective strengths and limitations. MS allows for in-depth characterization of metabolic profiles due to high mass resolution and accuracy, as it is able to resolve and identify thousands of small molecules in a sample with high sensitivity. However, it is labor intensive, placing limitations on study sample size. Nuclear magnetic resonance (NMR), on the other hand, is effective for metabolite quantification in large-scale epidemiological studies as it requires minimal sample preparation and processing, offering high throughput capabilities. While NMR is still less sensitive than MS, technological developments have greatly increased the resolution and sensitivity of NMR, making it an overall more accessible and cost-effective tool for metabolic analysis, and it has been broadly applied in large-scale epidemiologic studies [14]. Indeed, association studies on diabetes and cardiovascular disease (CVD) have shown similar outcomes when NMR was used compared to the traditional method of MS [15].

Several previous studies have identified metabolites associated with DR, mainly proliferative DR (PDR), in serum, plasma, or vitreous of patients with diabetes using MS [7,8,9,10,11,12]. The main metabolic pathways identified to be dysregulated in DR were amino acid metabolic pathways [10,11,12], as well as the pentose phosphate and galactose metabolism pathways [8]. In particular, arginine and proline were found to be associated with DR/PDR [10,11,12]. However, due to the constraints of mass spectrometry or the invasive nature of vitreous collection, these studies were limited by small case-control samples ranging from 20 to 173 samples, and in the majority of the studies, no adjustment for confounding factors was performed. In addition, metabolites in urine have recently been shown to provide information independent of serum or plasma metabolites. In the current study, we examined the cross-sectional association of DR with a combination of serum/plasma and urinary metabolites in three well-characterized cohorts in Singapore using NMR profiling.

## 2. Methods

### 2.1. Study Population

Data for this study were derived from Chinese, Malay, and Indian participants aged ≥40 years who participated in the baseline visit of three independent population-based studies in Singapore: the Singapore Malay Eye Study (SMES, 2004–2006, *n* = 3280), the Singapore Indian Eye Study (SINDI, 2007–2009, *n* = 3400), and the Singapore Chinese Eye Study (SCES, 2009–2011, *n* = 3353). The detailed recruitment and methodology of these studies have been published elsewhere [16,17]. All three studies followed the same methodology and were conducted in the same study clinic. All participants completed a questionnaire interview, underwent a standardized clinical and ocular examination including retinal photography and the collection of blood and urinary samples. The three studies were conducted in accordance to the tenets of the Declaration of Helsinki, and ethics approval was obtained from the Singapore Eye Research Institute Institutional Review Board. Written informed consent was provided by all participants.

For the current analysis, we included only those with diabetes (*n* = 592 Chinese, 1052 Malays, and 1320 Indians) and had their metabolites measured (*n* = 494 Chinese, 1025 Malays, and 1266 Indians for serum/plasma, and *n* = 527 Chinese and 1212 Indians for urine). Of these, after excluding those with ungradable retinal photographs or missing values for key covariates included in the multivariable model, 485 Chinese, 945 Malays, and 1213 Indians were included for the final serum/plasma metabolites analysis. Serum samples were collected from the Chinese and Indian participants, whilst plasma samples were collected for analysis from the Malay participants. The Malay cohort was not included in the analysis of urine metabolites as data on the urinary metabolites were not available for two thirds of the Malay cohort. Hence, for urinary metabolites, 509 Chinese and 1153 Indians with data on urinary metabolites and key covariates were included for the final analysis.

### 2.2. Assessment of Diabetes and Presence and Severity of DR

Diabetes was defined as random glucose ≥11.1 mmol/L or HbA1c ≥ 6.5% or participant reported physician-diagnosed diabetes, or the use of anti-diabetic medication. For the assessment of presence and severity of DR, participants were subject to pupil dilation, followed by 2-field color photographs for both eyes—Early Treatment for Diabetic Retinopathy Study (ETDRS) field 1 which is centered on the optic disc, and field 2 which is centered on the fovea. These photographs were taken using the Canon CR-1 Mark-II Non-mydriatic Digital Retina camera. DR was considered to be present if any characteristic lesions were noted on the retinal photographs, including microaneurysms, hemorrhages, cotton wool spots, intraretinal microvascular abnormalities, hard exudates, venous beading, and/or neovascularization. DR severity grading was conducted by trained retinal graders from the University of Sydney, Australia. Based on the modified Airlie House classification system, retinopathy severity was categorized as minimal (level 20), moderate (level 43–47), severe non-proliferative (level 53), and proliferative (level > 60) [14]. Taking the severity score of the worse eye into consideration, participants with ETDRS ≥ 20 were defined as having any DR. Moderate/above DR was defined as ETDRS ≥ 43, and vision threatening diabetic retinopathy (VTDR) as ETDRS ≥ 53 or the presence of clinically significant macular edema [18].

### 2.3. Assessment of Covariates

Information on age, sex, ethnicity, personal history of diabetes, duration of diabetes, and medication were collected using standardized questionnaires. Blood pressure (BP) measurements were taken using a digital automatic blood pressure monitor after the participant was seated for at least 5 minutes and an average of two measurements were taken as the blood pressure value for that individual. HbA1c and random blood glucose were assessed from blood samples.

### 2.4. Sample Collection, Storage, and Handling

Blood and urine samples for our study were collected at baseline and stored at −80 °C until time of metabolite analysis between 8 to 15 years after collection. The average time taken to process the samples from the time of collection to the time of storage was 4–6 h. The samples were subsequently stored at −80 °C until the time of analysis.

A number of studies have assessed the effect of storage of samples at −80 °C over various durations on metabolites [19,20,21,22,23,24]. A study by Hebels et al analyzed the metabolic profiles of plasma using ultra performance liquid chromatography time-of-flight mass spectrometry (UPLC-ToFMS) metabolomics, as well as transcriptomics and epigenomics (CpG methylation). They found that storage of samples at −80 °C for 13 to 17 years did not significantly alter the sample metabolic profiles [24]. In addition, they also noted that there was no significant difference between the freezing of plasma samples immediately and storage of the plasma at 4 °C for 8 h before freezing [24]. Three other studies using NMR analysis found that storage of samples at −80 °C over a period of 3 months [20], 10 months [21], and 30 months [19] did not significantly affect metabolomic profiles. With regard to urinary metabolites, in a study by Remer et al., 24-h urine samples were collected and stored at −22 °C. Urinary metabolites were quantified at baseline shortly after collection, and again after 12 or 15 years. The study found that 15 of 21 analytes measured (creatinine, urea, osmolality, iodine, nitrogen, anions, cations, acid-base parameters, and organic acids) were not significantly different from baseline after prolonged storage of more than 10 years [23].

### 2.5. Metabolite Quantification

A high-throughput NMR metabolomics platform (Nightingale Health, Helsinki, Finland) was used to quantify 228 metabolic measures from baseline serum/plasma samples and 54 metabolites from baseline urine samples. Serum/plasma metabolites included routine lipids, lipoprotein subclasses with lipid concentrations within 14 subclasses, fatty acid, amino acids, ketone bodies, and glycolysis related metabolites. The 14 lipoprotein subclasses include six subclasses of VLDL (extremely large, very large, large, medium, small, very small), IDL, three subclasses of LDL (large, medium, small), and four subclasses of HDL (very large, large, medium, small). Lipid concentration within each lipoprotein particle included triacylglycerol, total cholesterol, non-esterified cholesterol and cholesteryl ester levels, and phospholipid concentrations. Urine metabolites included amino acids, dietary, microbial, phenyl alanine, and pyrimidine. Metabolic measures were quantified, either as absolute concentrations of each metabolic measure or as ratios. Details of the NMR metabolomics quantification have been described previously [14].

### 2.6. Statistical Analyses

For analyses, metabolic concentrations below the detection limit were imputed by the minimum values detected for the corresponding metabolites. To alleviate the skewness of distributions, all metabolite concentrations were log_e_ (metabolite) transformed. To facilitate comparison of associations independent of the concentration ranges, we standardized each transformed concentration to mean 0, variance 1 for each cohort separately. Standardized values ≥4 or ≤−4 were considered outliers and thus excluded. Next, we implemented logistic regression adjusting for age, sex, duration of diabetes, HbA1c, and systolic blood pressure to estimate the odds ratio (OR) and 95% confidence interval (95% CI) for each metabolite in each cohort separately. Metabolites having *p*-values < 0.05 in at least one cohort and associations of the same direction in Chinese, Malay, and Indian cohorts for serum/plasma, or Chinese and Indian cohorts for urinary, were carried into meta-analysis, where results from individual cohorts were combined using generic inverse variance-weighted fixed effect model. The presence of heterogeneity was evaluated using $I^{2}$ statistic. Pooled results were reported as pooled OR (POR) with 95% CI. Statistical significance of the meta-analyzed *p*-values was pre-specified as 0.05/*n* using Bonferroni correction (BC) where *n* was the number of serum/plasma or urine metabolites passed to the meta-analysis accordingly. After BC, statistical significance for serum/plasma metabolites was considered at *p*-value < 0.00075, 0.00072, and 0.00161 for outcomes any DR, moderate/above DR, and VTDR respectively, while for urine metabolites it was <0.00714, 0.00417, and 0.005, respectively. Our approach to meta-analysis was similar to the methods adopted by recently published studies that analyzed NMR profiled metabolites associated with diabetes [19] and impaired kidney function [20]. Lastly, to evaluate if the addition of serum/plasma metabolites improves the prediction of DR beyond traditional risk factors, we plotted the area under the curve (AUC) of receiver operating characteristic (ROC) curves based on two multivariate logistic regression models: (1) only considered traditional risk factors (age, sex, ethnicity, duration of diabetes, hemoglobin A1c, and systolic blood pressure) (2) including variables in model 1, plus serum/plasma metabolites significant after BC. For prediction analyses, we did not include urinary metabolites, since it was not available in the Malay cohort. Meta-analysis was performed in Review Manager (RevMan) Version 5.3. (Copenhagen: The Nordic Cochrane Centre, The Cochrane Collaboration, 2014). Other statistical analyses were employed using R version 4.0.0 (R Foundation for Statistical Computing, Vienna, Austria; https://www.R-project.org/, accessed on 8 May 2020).

## 3. Results

### 3.1. Study Population

Participants with and without DR were not statistically different in age and gender (Table 1). Those with DR of all severities had longer durations of diabetes and higher HbA1c levels than those without DR (Table 1). Compared to participants with no DR (5.6 years), mean duration of diabetes was 13.1 years in those with any DR, 15.1 years in those with moderate/above DR, and 16.6 years in those with VTDR. Similarly, compared to those with no DR (7.5%), HbA1c levels were higher in those with any DR, moderate/above DR and VTDR (8.4%, 8.8%, and 8.7%). Participants with any DR, moderate/above DR, or VTDR were also noted to have significantly higher systolic BP than participants with no DR (mean systolic BP = 149.1 mm Hg, 154.4 mm Hg and 154.6 mm Hg vs. 144.3 mm Hg in those with no DR).

### 3.2. Results of NMR Detection of Metabolites in Serum/Plasma

**Figure 1:**
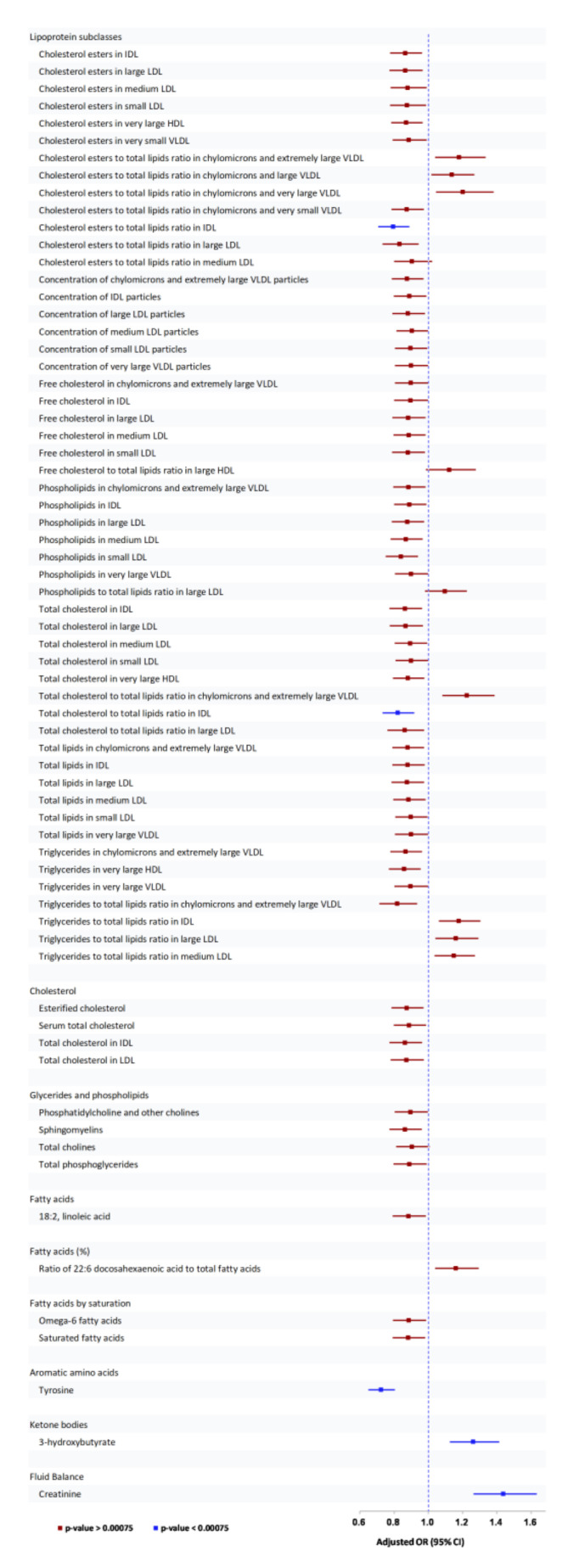
Association between serum/plasma metabolites and any DR in Chinese, Indians, and Malay cohorts. OR estimate corresponds to per SD ↑ in serum metabolites; OR estimates adjusted for age, sex, systolic BP, duration of diabetes, and HbA1c%.

In separate analysis of the three ethnic groups, 67 serum/plasma metabolites that were found to be associated with any DR at *p* < 0.05 were carried to meta-analysis. However, after accounting for multiple testing using BC, only five metabolites were significantly associated with any DR (Table S1). Similarly, in analyses for moderate/above DR, 69 serum/plasma metabolites were carried to meta-analysis, but after BC, only 13 serum/plasma metabolites were associated with moderate/above DR (Table S2). For outcome VTDR, 31 serum/plasma metabolites were significant at *p* < 0.05, of which nine serum/plasma metabolites were associated with VTDR (Table S3) after BC. Figure 1 shows the association of serum/plasma metabolites with any DR. Associations with moderate/above DR, and VTDR are shown in Supplementary Figures S1 and S2. Since the three cohorts have similar methodology and share similar environment, heterogeneity was acceptable or negligible. For example, for outcome any DR, $I^{2}$ for significant serum/plasma metabolites ranged from 0–29% whereas for urinary metabolites, $I^{2}$ was negligible (0%). Associations of specific metabolite group with outcomes are summarized below.

#### 3.2.1. Association of Serum/Plasma Metabolites with Any DR

Figure 1 shows the pooled ORs of 69 metabolites that were significant in the multivariable model and had similar direction of association with any DR in all three cohorts. In meta-analysis of the three cohorts, applying BC, 5 of the 69 metabolites were significantly associated with any DR with *p* values < 0.00075. The lipoprotein subclasses, cholesterol esters, and total cholesterol to total lipids ratio in IDL and amino acid tyrosine were protectively associated while 3-hydroxy butyrate (ketone) and creatinine were positively associated with any DR.

#### 3.2.2. Association of Serum/Plasma Metabolites with Moderate/above DR

In total, 13 metabolites were significantly associated with moderate/above DR after BC. Among the lipoprotein subclasses, ratios of cholesterol esters to total lipids in chylomicrons and extremely large VLDL, large VLDL and very large VLDL, and total cholesterol in chylomicrons and extremely large VLDL, were positively associated while ratios of cholesterol esters and total cholesterol to total lipids in IDL, phospholipids in small LDL, and total cholesterol to total lipids ratio in IDL and very large HDL were protectively associated with moderate/above DR (Figure S1 and Table 2). In addition, sphingomyelin, and ratio of saturated to total fatty acids, apolipoprotein A-1, and tyrosine were protectively associated while creatinine was positively associated with moderate/above DR.

#### 3.2.3. Association of Serum/Plasma Metabolites with VTDR

Nine metabolites were significantly associated with VTDR after BC. Among the lipoprotein subclasses, ratio of cholesterol esters to total lipids in chylomicrons and extremely large VLDL, ratio of total cholesterol to total lipids in chylomicrons and extremely large VLDL, and triglycerides in IDL were positively associated (Figure S3 and Table 2). Ratio of cholesterol esters to total lipids in IDL, ratio of triglycerides to total lipids in chylomicrons in extremely large VLDL, and ratio of SFA to TFA were protectively associated with VTDR (Figure S3 and Table 2). We also found that 3-hydroxybutyrate and creatinine were positively associated while tyrosine was protectively associated with VTDR (Figure S3 and Table 2).

### 3.3. Results of NMR Detection of Metabolites in Urinary Samples

Seven urinary metabolites were carried to meta-analysis for association with any DR, based on ethnic group analysis, of which five were found to be associated with any DR after BC (Table S4). Twelve urinary metabolites were carried to meta-analysis for association with moderate/above DR, of which eight were found to be associated with moderate/above DR after BC (Table S5). Ten urinary metabolites were carried to meta-analysis for association with VTDR, of which seven were found to be associated with moderate/above DR after BC (Table S6).

#### 3.3.1. Association of Urinary Metabolites with Any DR

**Figure 2:**
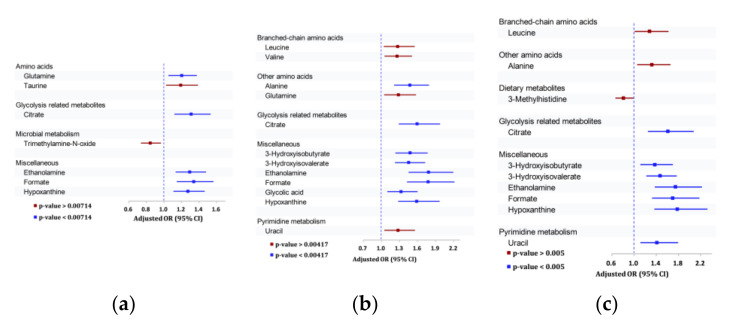
Association between urinary metabolites and (**a**) any DR, (**b**) moderate/above DR, and (**c**) vision-threatening DR in Chinese and Indians cohorts. OR estimate corresponds to per SD ↓ in urine metabolites; OR estimates adjusted for age, sex, systolic BP, duration of diabetes, and HbA1c%.

Figure 2 shows the pooled ORs of seven urinary metabolites that were associated with any DR. Of these, lower urinary levels of five of the seven metabolites were significantly associated with any DR outcomes, with *p*-values < 0.00714 (Table S4, Figure 2a). These included glutamine, citrate, ethanolamine, formate, and hypoxanthine.

#### 3.3.2. Association of Urinary Metabolites with Moderate/Severe DR outcomes

Figure 2b shows the pooled ORs of 12 urinary metabolites that were associated with Moderate/Severe DR Outcomes. Of these, lower urinary levels of alanine, citrate, 3-hydroxyisobutyrate, 3-hydroxyisovalerate, ethanolamine, formate, glycolic acid, and hypoxanthine were significantly associated with moderate/severe DR outcomes, with *p*-values < 0.00417 (Table S5, Figure 2b).

#### 3.3.3. Association of Urinary Metabolites with VTDR

Figure 2c shows the pooled ORs of 10 urinary metabolites that were associated with VTDR. Of these, lower urinary levels of citrate, 3-hydroxyisobutyrate, 3-hydroxyisovalerate, ethanolamine, formate, hypoxanthine, and uracil were significantly associated with VTDR, with *p*-values < 0.005 (Table S5, Figure 2c).

### 3.4. Serum/Plasma or Urinary Metabolites Associated with All Three Outcomes

Comparing the results from any DR, moderate/above DR, and VTDR, we identified three serum/plasma metabolites and four urine metabolites that were significantly associated with all three DR outcomes (Table 2). For all three DR outcomes, higher levels of serum/plasma tyrosine and cholesterol esters to total lipids ratio in IDL were identified to be inversely associated, while higher levels of serum/plasma creatinine were positively associated. Amongst the urinary metabolites, citrate, ethanolamine, formate, and hypoxanthine were inversely associated with all three DR outcomes.

### 3.5. Correlation between Serum/Plasma and Urinary Metabolites

Higher levels of serum/plasma 3-hydroxy butyrate (1.34 [1.13–1.60], 0.00097) and correspondingly lower urinary levels of 3-hydroxyisobutyrate (1.38 [1.11–1.70], 0.00295) were found to be associated with VTDR.

### 3.6. Receiver Operating Characteristic (ROC) Curve Analysis Comparing Traditional Risk Factors with the Addition of Serum and Urinary Metabolite Results

Finally, to evaluate whether the addition of serum/plasma metabolites improves prediction of DR, we plotted ROC curves and compared the AUC values between the two models. Compared to traditional risk factors alone, addition of serum metabolites to traditional risk factors significantly improved prediction for all three outcomes with AUC (95% CI) of 0.777 (0.756–0.798) vs. 0.795 (0.774–0.815) for traditional + serum/plasma metabolites (*p* < 0.001) for any DR; 0.824 (0.797–0.852) vs. 0.854 (0.829–0.879), *p* = 0.003 for moderate/above DR; 0.835 (0.803–0.867), vs. 0.868 (0.8441–0.895), *p* = 0.004 for VTDR. (Figure S3).

## 4. Discussion

In a population-based sample of Chinese, Malay, and Indian adults with diabetes, we found sixteen serum/plasma and ten urinary metabolites to be significantly associated with one of the three DR outcomes measured. The serum/plasma metabolites associated with at least one of the three DR outcomes were the amino acid tyrosine, saturated fatty acids, creatinine, Apolipoprotein A-I, 3-hydroxybutate (ketone bodies), sphingomyelins, and phospholipids in small LDL, cholesterol esters to total lipids ratio in IDL, chylomicrons and large/very large/extremely large VLDL, triglycerides to total lipids in IDL, chylomicrons and extremely large VLDL, and total cholesterol to total lipids ratio in chylomicrons and extremely large VLDL, IDL, and large HDL. The urinary metabolites associated with at least one of the three DR outcomes were citrate, ethanolamine, formate, hypoxanthine, 3-hydroxyisovalerate, 3-hydroxyisobutyrate, alanine, glutamine, uracil, and glycolic acid. We identified three serum/plasma metabolites and four urine metabolites that were significantly associated with all severities of DR: higher levels of tyrosine and higher cholesterol esters to total lipids ratio in IDL were found to be protective for all severities of DR, while higher levels of creatinine were found to be positively associated with all three DR outcomes. For urinary metabolites, higher levels of citrate, ethanolamine, formate, and hypoxanthine were negatively associated with all three DR outcomes. In ROC analyses, addition of significant serum/plasma metabolites modestly improved the prediction accuracy of any DR, moderate and above DR, and VTDR beyond traditional risk factors.

DR is a complex chronic metabolic disease, involving an interplay of various metabolic pathways. The pathophysiology of DR remains incomplete. Current understanding can be broadly discussed in three main aspects—hyperglycemia induced damage, inflammation, and retinal neurodegeneration.

Table 3 shows previous studies that have evaluated plasma, serum/plasma, and vitreous humor metabolites in patients with DR [7,8,9,10,11,12]. In previous metabolomic studies on DR, the majority of metabolites identified to be associated with DR were amino acids, including arginine, proline, and citrulline [10,11,12]. Of these studies, two analyzed vitreous samples with a focus on PDR as the outcome—they found that PDR was associated with increases in proline, arginine, ornithine, citrulline, lactose, and glucose, as well as with decreases in galactitol and ascorbic acid [7,10]. The other four studies analyzed serum/plasma or plasma, and found an association between DR and amino acid pathways [9,11,12], the pyrimidine metabolism pathway [12], and the pentose phosphate and galactose metabolism pathways [8]. These four studies also mainly focused on PDR as the main outcome.

Similar to the previous studies, our study found that lower serum/plasma levels of the amino acid tyrosine were positively associated with DR. This is in agreement with a previous hospital-based cross sectional study that found that low plasma tyrosine and phenylalanine levels were a risk factor for DR in patients with type 2 diabetes [25]. One aspect of the pathophysiology of DR is that of retinal neurodegeneration. It has been shown that in rats with diabetes, a deficiency in dopamine promoted neurodegeneration, resulting in early retinal dysfunction [26,27]. Dopamine is present in both the brain and the retina. It is derived from L-dihydroxyphenylalanine (L-DOPA), which is produced by hydroxylation of tyrosine. Hence, low levels of tyrosine may lead to a dopamine deficiency and thus promote neurodegeneration and early retinal dysfunction. Furthermore, a previous prospective study also found that having lower plasma tyrosine levels in patients with diabetes is associated with an increased risk of developing microvascular diseases [28]. It would be interesting to explore serum/plasma tyrosine as a biomarker for risk of developing DR in patients with diabetes. Tyrosine, being a commonly found dietary amino acid, can also be explored as a dietary supplement for therapeutic prevention of onset and progression of DR.

Serum/plasma creatinine is used clinically as a measurement of kidney function. Both nephropathy and retinopathy are known microvascular complications of diabetes. The prevalence of both of these complications is known to increase proportionally to the duration of type 2 DM [29,30]. A few studies have been done to study the association of DR and CKD. The Microalbuminuria Collaborative Study Group has reported that DR is not an independent predictor of albuminuria; however, other studies report that the presence and severity of DR is an indicator of the risk of developing renal dysfunction [31,32]. Therefore, our finding that increased serum/plasma creatinine levels are positively associated with DR may be a reflection of the concomitant development of microvascular complications of diabetes, diabetic nephropathy, and DR, with increasing DM duration or severity. As serum/plasma creatinine is a commonly tested component of the renal panel, clinically, it may be worth exploring and validating the use of serum/plasma creatinine as a biomarker for monitoring for DR progression. However, further studies will be required to determine if this finding reflects a parallel effect of diabetes on the development of DR and CKD, or if there exists a direct correlation between the disease processes of DR and CKD.

Several studies have been done to investigate the correlation between elevated serum/plasma cholesterol and lipid levels with DR. In particular, studies focusing on the treatment of hyperlipidemia have highlighted a possible correlation between serum/plasma cholesterol and lipid levels with DR. Our study noted that lower levels of serum/plasma cholesterol esters to total lipids ratio in IDL and higher levels of total cholesterol to total lipids ratio in chylomicrons and extremely large VLDL were significantly associated with moderate/above DR. Furthermore, lower levels of ratio of saturated fatty acids to total fatty were associated with VTDR. Previous studies have found that diabetes affects the synthesis and metabolism of triglyceride-rich lipoprotein particles [33]. However, the lipoprotein cascade is a continuous flux and VLDL assembly in the liver is affected by chylomicron changes, through the delivery of triglyceride and cholesterol, making it difficult to tease out the influence and effect on or of each component. One study analyzed the lipoprotein profile of 920 patients with and without T2DM, and found that patients with T2DM had increased concentration and size of smaller LDL particles [33]. Interestingly, in our study, an inverse correlation was observed between moderate/above DR and lipoproteins—moderate/above DR was significantly associated with lower levels of IDL and higher levels of the large/very large/extremely large extremely large VLDL, as compared to participants with no DR. This may suggest that this inversion in correlation in patients with DM may contribute to the development and hence pathophysiology of DR.

The association between serum/plasma saturated fatty acids and diabetes has not been conclusive. Most studies done on this subject so far support the role of odd-chain saturated fatty acids being protective and even-chain saturated fatty acids having an adverse effect [34,35,36,37,38,39], although further definitive work needs to be done to confirm this association. The possible difference in the effect of odd-chained and even-chained fatty acids on diabetes may explain the variation in association noted between lower levels of saturated FAs and the three DR groups studied—DR was only seen in participants with moderate/above DR and VTDR in our study. Further analysis by sub-quantification of odd-chained and even-chained fatty acid levels may provide more consistent results.

Few studies have been done to investigate urinary metabolites. Interestingly, our study identified significantly higher levels of serum/plasma 3-hydroxybutyrate in participants with DR and participants with VTDR, and correspondingly, lower levels of urinary 3-hydroxyisobutyrate in individuals with moderate and above DR, and VTDR. This suggests a correlation between serum/plasma and urinary levels of metabolites involved in ketone metabolism. Understanding of the physiological role of formate remains limited. It acts as an intermediary metabolite in folate-mediated one-carbon metabolism—a network of biochemical reactions essential for the biosynthesis of purines and generation of methyl groups [40]. It is potentially toxic and is excreted in the urine. Urinary formate concentrations were found to be elevated in folate and vitamin B12 deficiencies [41]. Our study has found the lower levels of urinary formate are associated with a higher risk of DR. It would be worth exploring the possibility that impaired formate excretion contributes to the pathophysiology of DR.

Past studies have shown that patients with chronic kidney disease have increased acid retention as estimated glomerular filtration rate (eGFR) decreases with disease progression [42]. Further studies have shown that a decrease in urinary citrate reflects this increased acid retention [43,44]. A previous study also found that a lower level of urinary citrate is a manifestation of insulin resistance [43,45]. Our observation that lower urinary citrate is associated with a higher risk of DR may be due to this association—lower urinary citrate correlates with worse kidney function, due to more severe or longer duration of diabetes and hence a higher risk of severe microvascular complications of diabetes—CKD and DR. Previous studies have shown that lower levels of urinary isovalerate and 3-hydroxyl isobutyrate were linked to mitochondrial dysfunction in patients with diabetic kidney disease [46]. However, understanding of urinary alanine, valine, 3-hydroxy-isovalerate, and 3-hydroxy isobutyrate remains limited and more research is required to explore and understand the protective association with DR that we observed in our study.

The major strength of the current study is quantifying metabolites in large population-based, well characterized and diverse ethnic cohorts and evaluating associations using a combination of both serum/plasma and urinary metabolites. Although previous metabolomic studies on DR employed mass spectrometry which is more sensitive than NMR profiling, they were limited by the small sample sizes of less than 100 patients and 100 controls, resulting in large variability in findings and difficulties making conclusive associations.

Our study has some limitations. Being a cross-sectional study, it does not provide a longitudinal picture, as a prospective study would be able to do. Furthermore, while our study identifies metabolites that are present in significantly different levels in participants with different severities of DR, it does not provide insight into the causal relationship between the metabolites and DR. In addition, we included only Chinese and Indian adults for the urinary metabolite analyses since urine samples were not available in the Malay cohort.

In conclusion, we identified sixteen serum metabolites and ten urinary metabolites to be associated with DR. If confirmed in future longitudinal analysis, adding detailed metabolic profiling to traditional risk factors could help in the formulation of disease prevention strategies for reducing the burden of DR.

## Figures and Tables

**Table 1:** Baseline characteristics of Chinese, Malay, and Indian cohorts stratified by DR status.

Variables	Any DR			Moderate/above DR			VTDR		
	No *n* = 2211	Yes *n* = 666	*p*-Value	No *n* = 2549	Yes *n* = 328	*p*-Value	No *n* = 2660	Yes *n* = 217	*p*-Value
Age, years	61.7 (10.2)	61.1 (9.1)	0.128	61.6 (10.1)	61.7 (8.5)	0.759	61.6 (10)	61.7 (8.3)	0.790
Gender, n (%)			0.207			0.076			0.050
Female	1077 (75.8)	343 (24.2)		1243 (87.5)	177 (12.5)		1299 (91.5)	121 (8.5)	
Male	1134 (77.8)	323 (22.2)		1306 (89.6)	151 (10.4)		1361 (93.4)	96 (6.6)	
Ethnicity, n (%)			<0.001			0.372			0.561
Chinese	460 (79.2)	121 (20.8)		516 (88.8)	65 (11.2)		542 (93.3)	39 (6.7)	
Malay	806 (80)	202 (20)		882 (87.5)	126 (12.5)		934 (92.7)	74 (7.3)	
Indian	945 (73.4)	343 (26.6)		1151 (89.4)	137 (10.6)		1184 (91.9)	104 (8.1)	
Diabetes duration, y	5.6 (7.9)	13.1 (10)	<0.001	6.4 (8.3)	15.1 (10.1)	<0.001	6.6 (8.4)	16.6 (10.2)	<0.001
HbA1c, %	7.5 (1.6)	8.4 (1.9)	<0.001	7.6 (1.6)	8.8 (2)	<0.001	7.6 (1.6)	8.7 (1.9)	<0.001
SBP, mm Hg	144.3 (21.1)	149.1 (23.7)	<0.001	144.2 (21.1)	154.4 (24.8)	<0.001	144.6 (21.2)	154.6 (26.5)	<0.001
Total Cholesterol, mmol/L	5.2 (1.2)	5.1 (1.4)	0.048	5.2 (1.2)	5.2 (1.5)	0.771	5.1 (1.2)	5.3 (1.6)	0.116
LDL Cholesterol, mmol/L	3.2 (1)	3 (1.1)	<0.001	3.2 (1)	3.1 (1.1)	0.088	3.2 (1)	3.2 (1.2)	0.720
HDL Cholesterol, mmol/L	1.1 (0.3)	1.2 (0.4)	0.559	1.1 (0.3)	1.2 (0.4)	0.271	1.1 (0.3)	1.2 (0.4)	0.505

Abbreviations: HbA1c, glycated hemoglobin; SBP, systolic blood pressure; LDL, low-density lipoprotein; HDL, high-density lipoprotein. Data presented are mean (standard deviation) or frequency (percentage); *p*-values represent the difference in characteristics by DR status based on Student’s *t*-Test or Chi-square test as appropriate for the variable.

**Table 2:** Serum/plasma and urinary metabolites associated with any DR, moderate/above DR, and VTDR after BC.

Serum/Plasma/Urinary Metabolites	Any DR	Moderate/above DR	VTDR
Serum/plasma metabolites (Per SD increase)			
Lipoprotein subclasses			
Cholesterol esters to total lipids ratio in chylomicrons and extremely large VLDL	-	1.42 (1.20–1.68)	1.47 (1.20–1.79)
Cholesterol esters to total lipids ratio in chylomicrons and large VLDL	-	1.36 (1.17–1.58)	-
Cholesterol esters to total lipids ratio in chylomicrons and very large VLDL	-	1.54 (1.28–1.85)	-
Cholesterol esters to total lipids ratio in IDL	0.79 (0.71–0.89)	0.68 (0.59–0.79)	0.76 (0.64–0.90)
Phospholipids in small LDL	-	0.74 (0.64–0.87)	-
Total cholesterol to total lipids ratio in chylomicrons and extremely large VLDL	-	1.45 (1.23–1.71)	1.45 (1.20–1.76)
Total cholesterol to total lipids ratio in IDL	0.82 (0.73–0.92)	0.72 (0.62–0.83)	-
Total cholesterol to total lipids ratio in very large HDL	-	0.75 (0.64–0.88)	-
Triglycerides to total lipids in chylomicrons and extremely large VLDL	-	-	0.68 (0.55–0.83)
Triglycerides in IDL	-	-	1.36 (1.14–1.61)
Glycerides and phospholipids			
Sphingomyelins	-	0.77 (0.67–0.89)	-
Apolipoproteins			
Apolipoprotein A-I	-	0.76 (0.65–0.88)	-
Fatty acid by saturation %			
Ratio of SFA to TFA- FA saturation %	-	0.76 (0.65–0.88)	0.71 (0.60–0.85)
Amino acids			
Tyrosine—Aromatic amino acid	0.72 (0.65–0.81)	0.69 (0.60–0.80)	0.70 (0.59–0.83)
Ketone bodies			
3-hydroxybutyrate—Ketone bodies	1.26 (1.13–1.41)	-	1.34 (1.13–1.60)
Fluid balance			
Creatinine	1.44 (1.26–1.63)	1.70 (1.45–2.01)	1.96 (1.63–2.37)
Urinary metabolites (Per SD decrease)			
Alanine—Amino acid	-	1.47 (1.21–1.79)	-
Glutamine—Amino acid	1.20 (1.05–1.38)	-	-
Citrate—Glycolysis	1.31 (1.12–1.53)	1.59 (1.29–1.97)	1.61 (1.25–2.08)
3-Hydroxyisobutyrate	-	1.48 (1.23–1.77)	1.38 (1.11–1.70)
3-Hydroxyisovalerate	-	1.45 (1.23–1.73)	1.47 (1.22–1.77)
Ethanolamine—Dietary	1.30 (1.13–1.48)	1.78 (1.45–2.19)	1.75 (1.38–2.22)
Formate—Microbial	1.34 (1.15–1.57)	1.78 (1.43–2.21)	1.70 (1.32–2.18)
Glycolic Acid	-	1.33 (1.10–1.60)	-
Hypoxanthine	1.27 (1.11–1.46)	1.59 (1.28–1.97)	1.78 (1.37–2.32)
Uracil-Pyrimidine metabolism	-	-	1.41 (1.11–1.79)

Note: Pooled results are expressed in terms of odds ratio with 95% confidence interval. Serum metabolites are pooled results of Chinese, Indian, and Malay cohorts. Urine metabolites are pooled results of Chinese and Indian cohorts. Selected metabolites are selected after applying Bonferroni correction. Highlighted metabolites are significantly associated with all three DR outcomes.

**Table 3:** Association between metabolites and DR in literature.

Author, Year	Study Population	Sample Type	Metabolites Platform	Results
Sumarriva et al. 2019 [11]	83 DR patients and 90 Diabetic control patients	Plasma	Untargeted High-resolution MS with liquid chromatography	In DR, arginine and citrulline-related pathways are dysregulated. In PDR vs. NPDR patients, fatty acid metabolism is changed.
Zhu et al. 2019 [12]	21 PDR patients, 21 controls with diabetes of >10 years but no DR	Serum	LC-MS	Impairment of metabolism of pyrimidines, arginine, and proline was associated with PDR. Fumaric acid, uridine, acetic acid, and cytidine were identified as potential PDR biomarkers.
Paris et al. 2016 [10]	9 PDR patients and 11 non-diabetic control subjects	Vitreous humor	Targeted MS (RPLC-MS and HILIC-MS)	In patients with PDR, there were increases in proline, arginine, ornithine, and citrulline.
Munipally et al. 2011 [9]	22 NPDR patients, 24 PDR patients, and 35 control subjects	Serum	HPLC	Increased kynurenine, kynurenic acid, and 3-hydroxykynurenine in NPDR and PDR—suggest a probable association of IDO and tryptophan metabolites with DR
Barba et al. 2010 [7]	22 patients with T1DM with PDR and 22 non-diabetic patients with a macula hole	Vitreous humor	1H NMR	Increased lactose and glucose and decreased galactitol and ascorbic acid in PDR patients.
Chen et al. 2016 [8]	40 DR patients and 40 Diabetic control patients; with further validation on an independent set of 40 DR patients, 40 Diabetic control patients, and 40 controls without diabetes	Plasma	Gas chromatography-MS	In DR, there was enrichment of the pentose phosphate pathway and galactose metabolism pathway.

Abbreviations: DR, diabetic retinopathy; MS, mass spectrometry; PDR, proliferative diabetic retinopathy; NPDR, non-proliferative diabetic retinopathy; LC-MS, liquid chromatography mass spectrometry; RPLC-MS, reversed phase liquid chromatography mass spectrometry; HIILIC-MS, hydrophilic interaction liquid chromatography mass spectrometry; HLPC, high performance liquid chromatography; IDO, indoleamine 2,3-dioxygenase; T1DM, type 1 diabetes mellitus; 1H NMR, proton nuclear magnetic resonance.

## Data Availability

As the study involves human participants, the data cannot be made freely available in the manuscript, the supplemental files, or a public repository due to ethical restrictions. Nevertheless, the data are available from the Singapore Eye Research Institutional Ethics Committee for researchers who meet the criteria for access to confidential data. Interested researchers can send data access requests to the Singapore Eye Research Institute using the following email address: seri@seri.com.sg.

## Supplementary Materials

The following are available online at https://www.mdpi.com/article/10.3390/metabo11090614/s1; Figure S1: Association between serum/plasma metabolites and moderate/above diabetic retinopathy in Chinese, Indians and Malay cohorts, Figure S2: Association between serum/plasma metabolites and vision-threatening diabetic retinopathy in Chinese, Indians and Malay cohorts, Figure S3: ROC curves—metabolites and (a) any DR (b) Moderate and above DR (c) VTDR. Table S1: Serum/plasma metabolites associated with any DR, Table S2: Serum/plasma metabolites associated with moderate and above DR, Table S3: Serum/plasma metabolites associated with VTDR, Table S4: Urinary metabolites associated with any DR, Table S5: Urinary metabolites associated with moderate and above DR, Table S6: Urinary metabolites associated with VTDR.
