# Supplementary material for: Novel Serum and Urinary Metabolites Associated with Diabetic Retinopathy in Three Asian Cohorts
Source: Metabolites. 2021 Sep 9;11(9):614. doi: 10.3390/metabo11090614 (PMC8467425; doi:10.3390/metabo11090614)
Supplement: Supplementary file 1 [file metabolites-11-00614-s001.zip › metabolites-1253706-supplementary.pdf]

## SUPPLEMENTARY MATERIAL

**Table S1.** Serum/plasma metabolites associated with any DR.

| Category of metabolites | Name of metabolytes | Chinese | | Malay | | Indian | | Pooled | | | After Bonferroni Correction |
|---|---|---|---|---|---|---|---|---|---|---|---|
| | | OR | 95%CI | OR | 95%CI | OR | 95%CI | OR | 95%CI | P - value | |
| Ketone bodies | 3-hydroxybutyrate | 1.51 | 1.14 - 2.01 | 1.16 | 0.95 - 1.41 | 1.26 | 1.07 - 1.48 | 1.26 | 1.13 - 1.41 | 0.00007 | select |
| Fluid Balance | Creatinine | 1.45 | 1.09 - 1.95 | 1.50 | 1.18 - 1.9 | 1.40 | 1.17 - 1.67 | 1.44 | 1.26 - 1.63 | 0.00000 | select |
| Fatty acids (%) | Ratio of 22:6 docosahexaenoic acid to total fatty acids | 1.02 | 0.79 - 1.31 | 1.11 | 0.92 - 1.34 | 1.26 | 1.08 - 1.48 | 1.16 | 1.04 - 1.29 | 0.00827 | |
| Cholesterol | Esterified cholesterol | 0.88 | 0.68 - 1.14 | 0.71 | 0.59 - 0.87 | 0.97 | 0.84 - 1.13 | 0.87 | 0.78 - 0.97 | 0.01294 | |
| Fatty acids by saturation | Omega-6 fatty acids | 0.80 | 0.61 - 1.05 | 0.77 | 0.63 - 0.93 | 0.99 | 0.86 - 1.16 | 0.88 | 0.79 - 0.99 | 0.02747 | |
| Lipoprotein subclasses & Cholesterol | Total cholesterol in IDL | 0.84 | 0.64 - 1.10 | 0.74 | 0.60 - 0.90 | 0.95 | 0.82 - 1.1 | 0.86 | 0.77 - 0.96 | 0.00921 | |
| Lipoprotein subclasses | Total cholesterol to total lipids ratio in IDL | 0.87 | 0.68 - 1.12 | 0.70 | 0.56 - 0.87 | 0.87 | 0.74 - 1.01 | 0.82 | 0.73 - 0.92 | 0.00056 | select |
| Lipoprotein subclasses | Cholesterol esters in IDL | 0.85 | 0.65 - 1.09 | 0.72 | 0.59 - 0.88 | 0.96 | 0.83 - 1.11 | 0.86 | 0.78 - 0.96 | 0.00839 | |
| Lipoprotein subclasses | Cholesterol esters to total lipids ratio in IDL | 0.75 | 0.58 - 0.98 | 0.69 | 0.56 - 0.86 | 0.86 | 0.74 - 1.01 | 0.79 | 0.71 - 0.89 | 0.00007 | select |
| Lipoprotein subclasses | Free cholesterol in IDL | 0.95 | 0.72 - 1.25 | 0.78 | 0.64 - 0.95 | 0.95 | 0.82 - 1.11 | 0.90 | 0.80 - 1.00 | 0.05121 | |
| Lipoprotein subclasses | Total lipids in IDL | 0.88 | 0.68 - 1.14 | 0.75 | 0.62 - 0.91 | 0.97 | 0.83 - 1.12 | 0.88 | 0.79 - 0.98 | 0.01879 | |
| Lipoprotein subclasses | Concentration of IDL particles | 0.87 | 0.67 - 1.12 | 0.78 | 0.65 - 0.95 | 0.97 | 0.83 - 1.13 | 0.89 | 0.80 - 0.99 | 0.03083 | |

| | | | | | | | | | | |
|---|---|---|---|---|---|---|---|---|---|---|
| Lipoprotein subclasses | Phospholipids in IDL | 0.91 | 0.71 - 1.17 | 0.75 | 0.62 - 0.91 | 0.97 | 0.84 - 1.13 | 0.89 | 0.80 - 0.99 | 0.02956 |
| Lipoprotein subclasses | Triglycerides to total lipids ratio in IDL | 1.07 | 0.84 - 1.37 | 1.33 | 1.10 - 1.61 | 1.13 | 0.98 - 1.30 | 1.18 | 1.06 - 1.30 | 0.00204 |
| Fatty acids | 18:2, linoleic acid | 0.82 | 0.63 - 1.07 | 0.77 | 0.63 - 0.93 | 0.99 | 0.85 - 1.16 | 0.88 | 0.79 - 0.99 | 0.02714 |
| Cholesterol | Total cholesterol in LDL | 0.88 | 0.67 - 1.16 | 0.71 | 0.58 - 0.87 | 0.97 | 0.84 - 1.13 | 0.87 | 0.78 - 0.97 | 0.01522 |
| Lipoprotein subclasses | Free cholesterol to total lipids ratio in large HDL | 1.12 | 0.83 - 1.52 | 1.28 | 1.01 - 1.62 | 1.04 | 0.87 - 1.24 | 1.12 | 0.99 - 1.28 | 0.08041 |
| Lipoprotein subclasses | Total cholesterol in large LDL | 0.86 | 0.65 - 1.13 | 0.71 | 0.57 - 0.87 | 0.96 | 0.83 - 1.12 | 0.87 | 0.78 - 0.97 | 0.01123 |
| Lipoprotein subclasses | Total cholesterol to total lipids ratio in large LDL | 0.88 | 0.61 - 1.29 | 0.68 | 0.54 - 0.86 | 0.95 | 0.81 - 1.11 | 0.86 | 0.76 - 0.97 | 0.01790 |
| Lipoprotein subclasses | Cholesterol esters in large LDL | 0.85 | 0.65 - 1.11 | 0.69 | 0.56 - 0.86 | 0.97 | 0.83 - 1.12 | 0.86 | 0.77 - 0.97 | 0.01115 |
| Lipoprotein subclasses | Cholesterol esters to total lipids ratio in large LDL | 0.79 | 0.58 - 1.08 | 0.62 | 0.48 - 0.80 | 0.95 | 0.81 - 1.12 | 0.83 | 0.73 - 0.94 | 0.00401 |
| Lipoprotein subclasses | Free cholesterol in large LDL | 0.91 | 0.68 - 1.22 | 0.75 | 0.62 - 0.91 | 0.96 | 0.83 - 1.11 | 0.88 | 0.79 - 0.98 | 0.02488 |
| Lipoprotein subclasses | Total lipids in large LDL | 0.86 | 0.66 - 1.12 | 0.72 | 0.59 - 0.88 | 0.98 | 0.84 - 1.13 | 0.88 | 0.78 - 0.98 | 0.01600 |
| Lipoprotein subclasses | Concentration of large LDL particles | 0.88 | 0.68 - 1.14 | 0.72 | 0.59 - 0.88 | 0.98 | 0.84 - 1.14 | 0.88 | 0.79 - 0.98 | 0.02035 |
| Lipoprotein subclasses | Phospholipids in large LDL | 0.87 | 0.67 - 1.13 | 0.75 | 0.61 - 0.90 | 0.97 | 0.83 - 1.12 | 0.88 | 0.79 - 0.97 | 0.01515 |
| Lipoprotein subclasses | Phospholipids to total lipids ratio in large LDL | 1.11 | 0.83 - 1.47 | 1.29 | 1.05 - 1.58 | 1.00 | 0.86 - 1.17 | 1.10 | 0.98 - 1.23 | 0.10656 |
| Lipoprotein subclasses | Triglycerides to total lipids ratio in large LDL | 1.05 | 0.80 - 1.37 | 1.34 | 1.11 - 1.62 | 1.10 | 0.94 - 1.27 | 1.16 | 1.04 - 1.29 | 0.00705 |

| | | | | | | | | | | |
|---|---|---|---|---|---|---|---|---|---|---|
| Lipoprotein subclasses | Cholesterol esters to total lipids ratio in chylomicrons and large VLDL | 1.14 | 0.85 - 1.51 | 1.29 | 1.05 - 1.58 | 1.06 | 0.91 - 1.23 | 1.14 | 1.02 - 1.27 | 0.02421 |
| Lipoprotein subclasses | Total cholesterol in medium LDL | 0.93 | 0.72 - 1.20 | 0.71 | 0.59 - 0.87 | 1.00 | 0.86 - 1.15 | 0.89 | 0.80 - 0.99 | 0.03888 |
| Lipoprotein subclasses | Cholesterol esters in medium LDL | 0.90 | 0.68 - 1.19 | 0.71 | 0.57 - 0.88 | 0.98 | 0.83 - 1.16 | 0.88 | 0.78 - 0.99 | 0.03447 |
| Lipoprotein subclasses | Cholesterol esters to total lipids ratio in medium LDL | 0.93 | 0.71 - 1.21 | 0.74 | 0.58 - 0.94 | 0.99 | 0.84 - 1.18 | 0.90 | 0.80 - 1.02 | 0.11005 |
| Lipoprotein subclasses | Free cholesterol in medium LDL | 0.89 | 0.69 - 1.16 | 0.76 | 0.63 - 0.91 | 0.97 | 0.84 - 1.12 | 0.88 | 0.80 - 0.98 | 0.02282 |
| Lipoprotein subclasses | Total lipids in medium LDL | 0.89 | 0.69 - 1.16 | 0.71 | 0.59 - 0.87 | 0.99 | 0.86 - 1.15 | 0.88 | 0.79 - 0.98 | 0.02378 |
| Lipoprotein subclasses | Concentration of medium LDL particles | 0.89 | 0.69 - 1.15 | 0.78 | 0.65 - 0.94 | 1.00 | 0.86 - 1.15 | 0.90 | 0.81 - 1.00 | 0.05714 |
| Lipoprotein subclasses | Phospholipids in medium LDL | 0.81 | 0.62 - 1.06 | 0.74 | 0.61 - 0.90 | 0.98 | 0.84 - 1.13 | 0.87 | 0.78 - 0.97 | 0.01047 |
| Lipoprotein subclasses | Triglycerides to total lipids ratio in medium LDL | 1.06 | 0.82 - 1.37 | 1.33 | 1.11 - 1.60 | 1.08 | 0.93 - 1.24 | 1.15 | 1.04 - 1.27 | 0.00860 |
| Glycerides and phospholipids | Phosphatidylcholine and other cholines | 0.84 | 0.65 - 1.08 | 0.79 | 0.65 - 0.96 | 0.98 | 0.85 - 1.14 | 0.90 | 0.80 - 1.00 | 0.04394 |
| Cholesterol | Serum total cholesterol | 0.86 | 0.67 - 1.11 | 0.76 | 0.62 - 0.92 | 0.98 | 0.85 - 1.14 | 0.89 | 0.80 - 0.99 | 0.02697 |
| Fatty acids by saturation | Saturated fatty acids | 0.76 | 0.58 - 1.00 | 0.78 | 0.65 - 0.95 | 0.99 | 0.85 - 1.15 | 0.88 | 0.79 - 0.98 | 0.02238 |
| Lipoprotein subclasses | Total cholesterol in small LDL | 0.95 | 0.74 - 1.23 | 0.72 | 0.59 - 0.87 | 1.00 | 0.86 - 1.15 | 0.90 | 0.81 - 1.00 | 0.04639 |
| Lipoprotein subclasses | Cholesterol esters in small LDL | 0.91 | 0.67 - 1.24 | 0.73 | 0.59 - 0.90 | 0.97 | 0.82 - 1.15 | 0.88 | 0.78 - 0.99 | 0.03090 |
| Lipoprotein subclasses | Free cholesterol in small LDL | 0.92 | 0.71 - 1.18 | 0.68 | 0.55 - 0.85 | 0.97 | 0.84 - 1.13 | 0.88 | 0.79 - 0.98 | 0.02148 |

| | | | | | | | | | | | |
|---|---|---|---|---|---|---|---|---|---|---|---|
| Lipoprotein subclasses | Total lipids in small LDL | 0.89 | 0.69 - 1.15 | 0.78 | 0.65 - 0.93 | 0.98 | 0.85 - 1.14 | 0.90 | 0.81 - 1.00 | 0.04046 | |
| Lipoprotein subclasses | Concentration of small LDL particles | 0.88 | 0.68 - 1.14 | 0.78 | 0.65 - 0.93 | 0.98 | 0.85 - 1.14 | 0.89 | 0.81 - 0.99 | 0.03836 | |
| Lipoprotein subclasses | Phospholipids in small LDL | 0.82 | 0.63 - 1.06 | 0.65 | 0.53 - 0.81 | 0.95 | 0.82 - 1.11 | 0.84 | 0.75 - 0.94 | 0.00219 | |
| Glycerides and phospholipids | Sphingomyelins | 0.86 | 0.67 - 1.11 | 0.76 | 0.63 - 0.92 | 0.93 | 0.80 - 1.09 | 0.86 | 0.77 - 0.96 | 0.00782 | |
| Glycerides and phospholipids | Total cholines | 0.84 | 0.65 - 1.08 | 0.81 | 0.66 - 0.98 | 0.99 | 0.85 - 1.15 | 0.90 | 0.81 - 1.01 | 0.06736 | |
| Glycerides and phospholipids | Total phosphoglycerides | 0.81 | 0.62 - 1.05 | 0.77 | 0.63 - 0.94 | 1.00 | 0.86 - 1.16 | 0.89 | 0.80 - 0.99 | 0.03292 | |
| Aromatic amino acids | Tyrosine | 0.70 | 0.54 - 0.91 | 0.72 | 0.59 - 0.87 | 0.73 | 0.63 - 0.85 | 0.72 | 0.65 - 0.81 | 0.00000 | select |
| Lipoprotein subclasses | Total cholesterol in very large HDL | 0.85 | 0.67 - 1.08 | 0.97 | 0.79 - 1.20 | 0.85 | 0.74 - 0.98 | 0.88 | 0.79 - 0.98 | 0.01618 | |
| Lipoprotein subclasses | Cholesterol esters in very large HDL | 0.85 | 0.67 - 1.07 | 0.95 | 0.76 - 1.18 | 0.85 | 0.74 - 0.97 | 0.87 | 0.78 - 0.97 | 0.00937 | |
| Lipoprotein subclasses | Triglycerides in very large HDL | 0.81 | 0.64 - 1.02 | 0.92 | 0.71 - 1.19 | 0.86 | 0.75 - 0.99 | 0.86 | 0.77 - 0.96 | 0.00546 | |
| Lipoprotein subclasses | Cholesterol esters to total lipids ratio in chylomicrons and very large VLDL | 1.21 | 0.85 - 1.72 | 1.42 | 1.13 - 1.77 | 1.04 | 0.84 - 1.28 | 1.20 | 1.04 - 1.38 | 0.01041 | |
| Lipoprotein subclasses | Total lipids in very large VLDL | 0.83 | 0.65 - 1.05 | 0.79 | 0.64 - 0.97 | 0.99 | 0.85 - 1.15 | 0.90 | 0.81 - 1.00 | 0.05325 | |
| Lipoprotein subclasses | Concentration of very large VLDL particles | 0.83 | 0.65 - 1.05 | 0.78 | 0.63 - 0.97 | 0.99 | 0.86 - 1.16 | 0.90 | 0.81 - 1.00 | 0.05364 | |
| Lipoprotein subclasses | Phospholipids in very large VLDL | 0.84 | 0.66 - 1.06 | 0.77 | 0.62 - 0.96 | 1.00 | 0.86 - 1.16 | 0.90 | 0.81 - 1.00 | 0.05576 | |

| | | | | | | | | | | |
|---|---|---|---|---|---|---|---|---|---|---|
| Lipoprotein subclasses | Triglycerides in very large VLDL | 0.83 | 0.65 - 1.05 | 0.79 | 0.63 - 0.99 | 0.98 | 0.84 - 1.13 | 0.90 | 0.80 - 1.00 | 0.04821 |
| Lipoprotein subclasses | Cholesterol esters in very small VLDL | 0.83 | 0.64 - 1.07 | 0.79 | 0.63 - 0.98 | 0.96 | 0.82 - 1.12 | 0.89 | 0.79 - 0.99 | 0.03451 |
| Lipoprotein subclasses | Cholesterol esters to total lipids ratio in chylomicrons and very small VLDL | 0.88 | 0.67 - 1.14 | 0.91 | 0.74 - 1.11 | 0.86 | 0.74 - 0.99 | 0.87 | 0.78 - 0.97 | 0.01460 |
| Lipoprotein subclasses | Total cholesterol to total lipids ratio in chylomicrons and extremely large VLDL | 1.11 | 0.82 - 1.51 | 1.19 | 0.94 - 1.51 | 1.28 | 1.08 - 1.51 | 1.22 | 1.08 - 1.39 | 0.00140 |
| Lipoprotein subclasses | Cholesterol esters to total lipids ratio in chylomicrons and extremely large VLDL | 1.04 | 0.79 - 1.38 | 1.22 | 0.96 - 1.56 | 1.21 | 1.02 - 1.44 | 1.18 | 1.04 - 1.33 | 0.00999 |
| Lipoprotein subclasses | Free cholesterol in chylomicrons and extremely large VLDL | 0.83 | 0.65 - 1.06 | 0.80 | 0.65 - 0.99 | 0.98 | 0.84 - 1.14 | 0.90 | 0.81 - 1.00 | 0.04830 |
| Lipoprotein subclasses | Total lipids in chylomicrons and extremely large VLDL | 0.79 | 0.62 - 1.00 | 0.82 | 0.68 - 0.99 | 0.95 | 0.82 - 1.11 | 0.88 | 0.79 - 0.98 | 0.01581 |
| Lipoprotein subclasses | Concentration of chylomicrons and extremely large VLDL particles | 0.78 | 0.61 - 1.00 | 0.82 | 0.68 - 0.99 | 0.95 | 0.82 - 1.10 | 0.87 | 0.79 - 0.97 | 0.01305 |
| Lipoprotein subclasses | Phospholipids in chylomicrons and extremely large VLDL | 0.82 | 0.64 - 1.04 | 0.81 | 0.67 - 0.99 | 0.95 | 0.82 - 1.11 | 0.88 | 0.79 - 0.98 | 0.02339 |
| Lipoprotein subclasses | Triglycerides in chylomicrons and extremely large VLDL | 0.78 | 0.61 - 0.99 | 0.80 | 0.66 - 0.98 | 0.94 | 0.81 - 1.09 | 0.87 | 0.78 - 0.96 | 0.00827 |
| Lipoprotein subclasses | Triglycerides to total lipids ratio in chylomicrons and extremely large VLDL | 0.98 | 0.63 - 1.52 | 0.91 | 0.74 - 1.12 | 0.72 | 0.60 - 0.88 | 0.82 | 0.71 - 0.94 | 0.00336 |

**Table S2. Serum/plasma metabolites associated with moderate and above DR**

| Category of metabolites | Name of metabolytes | Chinese | | Malay | | Indian | | Pooled | | | After Bonferroni Correction |
|---|---|---|---|---|---|---|---|---|---|---|---|
| | | OR | 95%CI | OR | 95%CI | OR | 95%CI | OR | 95%CI | P - value | |
| Apolipoproteins | Apolipoprotein A-I | 0.74 | 0.52 - 1.05 | 0.63 | 0.49 - 0.82 | 0.88 | 0.70 - 1.09 | 0.76 | 0.65 - 0.88 | 0.00033 | select |
| Apolipoproteins | Apolipoprotein B | 0.90 | 0.63 - 1.28 | 0.71 | 0.56 - 0.90 | 0.96 | 0.78 - 1.19 | 0.85 | 0.73 - 0.98 | 0.02565 | |
| Ketone bodies | 3-hydroxybutyrate | 1.91 | 1.30 - 2.81 | 1.16 | 0.92 - 1.48 | 1.10 | 0.87 - 1.38 | 1.22 | 1.05 - 1.42 | 0.00868 | |
| Fluid Balance | Creatinine | 1.68 | 1.14 - 2.47 | 1.44 | 1.09 - 1.89 | 1.95 | 1.53 - 2.48 | 1.70 | 1.45 - 2.01 | 0.00000 | select |
| Cholesterol | Esterified cholesterol | 0.96 | 0.67 - 1.36 | 0.60 | 0.47 - 0.77 | 0.90 | 0.74 - 1.11 | 0.79 | 0.69 - 0.92 | 0.00177 | |
| Fatty acids by saturation (%) | Ratio of omega-3 fatty acids to total fatty acids | 1.00 | 0.71 - 1.43 | 1.24 | 0.97 - 1.57 | 1.27 | 1.02 - 1.59 | 1.21 | 1.04 - 1.40 | 0.01223 | |
| Fatty acids by saturation | Omega-6 fatty acids | 0.97 | 0.67 - 1.39 | 0.67 | 0.53 - 0.85 | 0.89 | 0.72 - 1.10 | 0.81 | 0.70 - 0.94 | 0.00498 | |
| Cholesterol | Free cholesterol | 0.96 | 0.67 - 1.36 | 0.73 | 0.58 - 0.92 | 0.96 | 0.78 - 1.18 | 0.87 | 0.75 - 1.00 | 0.04740 | |
| Glycolysis related metabolites | Glucose | 1.43 | 0.93 - 2.19 | 1.59 | 1.14 - 2.20 | 1.16 | 0.92 - 1.47 | 1.32 | 1.10 - 1.57 | 0.00211 | |
| Cholesterol | Total cholesterol in HDL2 | 0.80 | 0.53 - 1.20 | 0.76 | 0.59 - 0.97 | 0.94 | 0.75 - 1.17 | 0.84 | 0.72 - 0.98 | 0.02798 | |
| Cholesterol | Total cholesterol in HDL3 | 0.96 | 0.67 - 1.39 | 0.63 | 0.49 - 0.81 | 0.98 | 0.79 - 1.21 | 0.84 | 0.72 - 0.97 | 0.02123 | |
| Cholesterol | Total cholesterol in HDL | 0.80 | 0.56 - 1.13 | 0.74 | 0.58 - 0.95 | 0.90 | 0.73 - 1.12 | 0.82 | 0.71 - 0.95 | 0.00953 | |
| Lipoprotein subclasses & Cholesterol | Total cholesterol in IDL | 1.00 | 0.69 - 1.44 | 0.66 | 0.51 - 0.85 | 0.90 | 0.72 - 1.11 | 0.82 | 0.71 - 0.95 | 0.00843 | |
| Lipoprotein subclasses | Total cholesterol to total lipids ratio in IDL | 0.94 | 0.68 - 1.31 | 0.58 | 0.44 - 0.76 | 0.73 | 0.59 - 0.90 | 0.72 | 0.62 - 0.83 | 0.00001 | select |
| Lipoprotein subclasses | Cholesterol esters in IDL | 0.98 | 0.69 - 1.40 | 0.63 | 0.49 - 0.81 | 0.90 | 0.73 - 1.11 | 0.81 | 0.70 - 0.94 | 0.00429 | |

| | | | | | | | | | | | |
|---|---|---|---|---|---|---|---|---|---|---|---|
| Lipoprotein subclasses | Cholesterol esters to total lipids ratio in IDL | 0.80 | 0.57 - 1.13 | 0.57 | 0.43 - 0.74 | 0.72 | 0.58 - 0.89 | 0.68 | 0.59 - 0.79 | 0.00000 | select |
| Fatty acids | 18:2, linoleic acid | 0.99 | 0.69 - 1.43 | 0.68 | 0.54 - 0.86 | 0.92 | 0.74 - 1.14 | 0.83 | 0.72 - 0.96 | 0.01136 | |
| Lipoprotein subclasses | Total cholesterol in large VLDL | 0.76 | 0.55 - 1.04 | 0.75 | 0.58 - 0.96 | 0.99 | 0.80 - 1.22 | 0.86 | 0.74 - 0.99 | 0.03280 | |
| Lipoprotein subclasses | Cholesterol esters to total lipids ratio in chylomicrons and large VLDL | 1.31 | 0.89 - 1.92 | 1.57 | 1.22 - 2.03 | 1.25 | 1.02 - 1.54 | 1.36 | 1.17 - 1.58 | 0.00005 | select |
| Lipoprotein subclasses | Free cholesterol in large VLDL | 0.75 | 0.55 - 1.03 | 0.71 | 0.54 - 0.93 | 0.99 | 0.81 - 1.23 | 0.85 | 0.73 - 0.98 | 0.02801 | |
| Lipoprotein subclasses | Free cholesterol to total lipids ratio in chylomicrons and large VLDL | 0.61 | 0.40 - 0.94 | 0.66 | 0.50 - 0.88 | 0.97 | 0.74 - 1.28 | 0.77 | 0.64 - 0.92 | 0.00390 | |
| Lipoprotein subclasses | Total lipids in large VLDL | 0.76 | 0.55 - 1.05 | 0.71 | 0.55 - 0.91 | 0.95 | 0.77 - 1.18 | 0.83 | 0.72 - 0.96 | 0.01039 | |
| Lipoprotein subclasses | Concentration of large VLDL particles | 0.76 | 0.55 - 1.06 | 0.71 | 0.55 - 0.91 | 0.95 | 0.77 - 1.17 | 0.83 | 0.72 - 0.95 | 0.00973 | |
| Lipoprotein subclasses | Phospholipids in large VLDL | 0.76 | 0.55 - 1.05 | 0.77 | 0.61 - 0.97 | 0.96 | 0.78 - 1.19 | 0.85 | 0.74 - 0.98 | 0.02092 | |
| Lipoprotein subclasses | Triglycerides in large VLDL | 0.76 | 0.55 - 1.06 | 0.70 | 0.54 - 0.90 | 0.95 | 0.77 - 1.17 | 0.82 | 0.71 - 0.95 | 0.00802 | |
| Lipoprotein subclasses | Total cholesterol in medium HDL | 0.83 | 0.59 - 1.18 | 0.72 | 0.56 - 0.92 | 0.93 | 0.74 - 1.15 | 0.83 | 0.71 - 0.96 | 0.01286 | |
| Lipoprotein subclasses | Cholesterol esters in medium HDL | 0.81 | 0.57 - 1.16 | 0.71 | 0.56 - 0.91 | 0.91 | 0.73 - 1.14 | 0.81 | 0.70 - 0.95 | 0.00712 | |
| Lipoprotein subclasses | Total lipids in medium HDL | 0.79 | 0.55 - 1.13 | 0.70 | 0.55 - 0.90 | 0.97 | 0.78 - 1.20 | 0.84 | 0.72 - 0.97 | 0.01633 | |

| | | | | | | | | | | | |
|---|---|---|---|---|---|---|---|---|---|---|---|
| Lipoprotein subclasses | Concentration of medium HDL particles | 0.77 | 0.54 - 1.11 | 0.70 | 0.55 - 0.90 | 0.97 | 0.79 - 1.20 | 0.84 | 0.72 - 0.97 | 0.01624 | |
| Lipoprotein subclasses | Phospholipids in medium HDL | 0.74 | 0.53 - 1.04 | 0.71 | 0.56 - 0.91 | 0.99 | 0.80 - 1.23 | 0.84 | 0.73 - 0.97 | 0.01901 | |
| Lipoprotein subclasses | Phospholipids in medium LDL | 0.95 | 0.66 - 1.37 | 0.66 | 0.52 - 0.83 | 0.90 | 0.73 - 1.12 | 0.81 | 0.70 - 0.94 | 0.00411 | |
| Lipoprotein subclasses | Triglycerides in medium VLDL | 0.81 | 0.58 - 1.13 | 0.79 | 0.63 - 1.00 | 0.95 | 0.76 - 1.17 | 0.86 | 0.74 - 0.99 | 0.03507 | |
| Glycerides and phospholipids | Phosphatidylcholine and other cholines | 0.81 | 0.57 - 1.15 | 0.67 | 0.53 - 0.86 | 0.92 | 0.74 - 1.14 | 0.80 | 0.69 - 0.93 | 0.00302 | |
| Fatty acids by saturation | Polyunsaturated fatty acids | 0.95 | 0.66 - 1.36 | 0.69 | 0.55 - 0.88 | 0.94 | 0.77 - 1.16 | 0.85 | 0.73 - 0.98 | 0.02284 | |
| Cholesterol | Remnant cholesterol (non-HDL, non-LDL -cholesterol) | 0.89 | 0.63 - 1.27 | 0.72 | 0.57 - 0.91 | 0.99 | 0.80 - 1.23 | 0.86 | 0.75 - 1.00 | 0.04373 | |
| Cholesterol | Serum total cholesterol | 0.94 | 0.66 - 1.33 | 0.66 | 0.52 - 0.83 | 0.92 | 0.75 - 1.13 | 0.82 | 0.71 - 0.94 | 0.00568 | |
| Fatty acids by saturation | Saturated fatty acids | 0.83 | 0.58 - 1.19 | 0.65 | 0.51 - 0.83 | 0.90 | 0.73 - 1.12 | 0.79 | 0.68 - 0.92 | 0.00182 | |
| Fatty acids by saturation (%) | Ratio of saturated fatty acids to total fatty acids | 0.69 | 0.47 - 1.00 | 0.81 | 0.64 - 1.03 | 0.74 | 0.59 - 0.91 | 0.76 | 0.65 - 0.88 | 0.00019 | select |
| Lipoprotein subclasses | Free cholesterol in small HDL | 0.80 | 0.56 - 1.15 | 0.76 | 0.60 - 0.96 | 0.93 | 0.75 - 1.16 | 0.84 | 0.72 - 0.97 | 0.01718 | |
| Lipoprotein subclasses | Total lipids in small HDL | 0.94 | 0.68 - 1.31 | 0.64 | 0.50 - 0.82 | 0.95 | 0.77 - 1.17 | 0.83 | 0.72 - 0.96 | 0.01057 | |
| Lipoprotein subclasses | Concentration of small HDL particles | 0.93 | 0.67 - 1.29 | 0.66 | 0.52 - 0.86 | 0.95 | 0.77 - 1.18 | 0.84 | 0.73 - 0.97 | 0.01975 | |

| | | | | | | | | | | | |
|---|---|---|---|---|---|---|---|---|---|---|---|
| Lipoprotein subclasses | Phospholipids in small HDL | 0.77 | 0.53 - 1.11 | 0.78 | 0.62 - 0.99 | 0.95 | 0.76 - 1.18 | 0.85 | 0.74 - 0.99 | 0.03276 | |
| Lipoprotein subclasses | Phospholipids in small LDL | 0.90 | 0.63 - 1.29 | 0.52 | 0.39 - 0.69 | 0.85 | 0.69 - 1.05 | 0.74 | 0.64 - 0.87 | 0.00013 | select |
| Glycerides and phospholipids | Sphingomyelins | 0.98 | 0.69 - 1.39 | 0.66 | 0.51 - 0.84 | 0.80 | 0.64 - 1.00 | 0.77 | 0.67 - 0.89 | 0.00058 | select |
| Glycerides and phospholipids | Total cholines | 0.84 | 0.59 - 1.20 | 0.69 | 0.54 - 0.88 | 0.92 | 0.74 - 1.14 | 0.82 | 0.71 - 0.94 | 0.00649 | |
| Total fatty acids and saturation measures | Total fatty acids | 0.87 | 0.61 - 1.25 | 0.67 | 0.53 - 0.86 | 0.95 | 0.77 - 1.18 | 0.83 | 0.72 - 0.96 | 0.01125 | |
| Glycerides and phospholipids | Total phosphoglycerides | 0.79 | 0.55 - 1.12 | 0.65 | 0.51 - 0.83 | 0.92 | 0.74 - 1.14 | 0.79 | 0.68 - 0.92 | 0.00172 | |
| Aromatic amino acids | Tyrosine | 0.57 | 0.40 - 0.82 | 0.67 | 0.53 - 0.85 | 0.76 | 0.62 - 0.93 | 0.69 | 0.60 - 0.80 | 0.00000 | select |
| Total fatty acids and saturation measures | Estimated degree of unsaturation | 1.14 | 0.80- 1.62 | 1.38 | 1.10 - 1.74 | 1.23 | 0.98 - 1.54 | 1.27 | 1.10 - 1.47 | 0.00138 | |
| Lipoprotein subclasses | Total cholesterol in very large HDL | 0.89 | 0.65 - 1.22 | 0.75 | 0.59 - 0.96 | 0.82 | 0.68 - 0.99 | 0.81 | 0.71 - 0.93 | 0.00273 | |
| Lipoprotein subclasses | Total cholesterol to total lipids ratio in very large HDL | 0.78 | 0.53 - 1.15 | 0.73 | 0.57 - 0.93 | 0.76 | 0.60 - 0.98 | 0.75 | 0.64 - 0.88 | 0.00044 | select |
| Lipoprotein subclasses | Cholesterol esters in very large HDL | 0.90 | 0.65 - 1.23 | 0.73 | 0.56 - 0.94 | 0.81 | 0.67 - 0.98 | 0.80 | 0.70 - 0.92 | 0.00177 | |
| Lipoprotein subclasses | Cholesterol esters to total lipids ratio in very large HDL | 0.85 | 0.58 - 1.26 | 0.74 | 0.58 - 0.95 | 0.76 | 0.59 - 0.98 | 0.77 | 0.65 - 0.90 | 0.00125 | |

| | | | | | | | | | | | |
|---|---|---|---|---|---|---|---|---|---|---|---|
| Lipoprotein subclasses | Free cholesterol to total lipids ratio in very large HDL | 0.70 | 0.45 - 1.08 | 0.75 | 0.58 - 0.97 | 0.81 | 0.64 - 1.03 | 0.77 | 0.66 - 0.91 | 0.00161 | |
| Lipoprotein subclasses | Phospholipids to total lipids ratio in very large HDL | 1.13 | 0.69 - 1.86 | 1.31 | 0.97 - 1.76 | 1.48 | 1.06 - 2.08 | 1.34 | 1.09 - 1.64 | 0.00537 | |
| Lipoprotein subclasses | Cholesterol esters to total lipids ratio in chylomicrons and very large VLDL | 1.46 | 0.91 - 2.33 | 1.72 | 1.30 - 2.28 | 1.38 | 1.03 - 1.85 | 1.54 | 1.28 - 1.85 | 0.00001 | select |
| Lipoprotein subclasses | Total lipids in very large VLDL | 0.74 | 0.54 - 1.00 | 0.68 | 0.53 - 0.88 | 0.95 | 0.77 - 1.16 | 0.81 | 0.70 - 0.94 | 0.00416 | |
| Lipoprotein subclasses | Concentration of very large VLDL particles | 0.74 | 0.54 - 1.00 | 0.68 | 0.52 - 0.88 | 0.95 | 0.77 - 1.16 | 0.81 | 0.70 - 0.93 | 0.00393 | |
| Lipoprotein subclasses | Phospholipids in very large VLDL | 0.75 | 0.55 - 1.03 | 0.66 | 0.51 - 0.86 | 0.97 | 0.79 - 1.19 | 0.82 | 0.71 - 0.94 | 0.00531 | |
| Lipoprotein subclasses | Phospholipids to total lipids ratio in chylomicrons and very large VLDL | 0.54 | 0.31 - 0.96 | 0.70 | 0.52 - 0.95 | 0.96 | 0.70 - 1.32 | 0.77 | 0.63 - 0.95 | 0.01471 | |
| Lipoprotein subclasses | Triglycerides in very large VLDL | 0.74 | 0.54 - 1.00 | 0.68 | 0.52 - 0.91 | 0.90 | 0.73 - 1.10 | 0.80 | 0.69 - 0.92 | 0.00231 | |
| Lipoprotein subclasses | Total cholesterol to total lipids ratio in chylomicrons and extremely large VLDL | 1.19 | 0.76 - 1.86 | 1.42 | 1.05 - 1.92 | 1.54 | 1.24 - 1.93 | 1.45 | 1.23 - 1.71 | 0.00001 | select |
| Lipoprotein subclasses | Cholesterol esters to total lipids ratio in chylomicrons and extremely large VLDL | 1.00 | 0.68 - 1.49 | 1.56 | 1.14 - 2.13 | 1.52 | 1.20 - 1.92 | 1.42 | 1.20 - 1.68 | 0.00005 | select |
| Lipoprotein subclasses | Free cholesterol in chylomicrons and extremely large VLDL | 0.75 | 0.55 - 1.02 | 0.73 | 0.57 - 0.93 | 0.94 | 0.77 - 1.16 | 0.83 | 0.72 - 0.95 | 0.00803 | |
| Lipoprotein subclasses | Total lipids in chylomicrons and extremely large VLDL | 0.74 | 0.54 - 1.02 | 0.74 | 0.59 - 0.93 | 0.94 | 0.76 - 1.15 | 0.83 | 0.72 - 0.95 | 0.00664 | |
| Lipoprotein subclasses | Concentration of chylomicrons and extremely large VLDL particles | 0.74 | 0.54 - 1.02 | 0.74 | 0.58 - 0.93 | 0.93 | 0.76 - 1.14 | 0.82 | 0.71 - 0.94 | 0.00545 | |

| | | | | | | | | | | |
|---|---|---|---|---|---|---|---|---|---|---|
| Lipoprotein subclasses | Phospholipids in chylomicrons and extremely large VLDL | 0.75 | 0.55 - 1.03 | 0.73 | 0.57 - 0.93 | 0.92 | 0.75 - 1.13 | 0.82 | 0.71 - 0.94 | 0.00569 |
| Lipoprotein subclasses | Phospholipids to total lipids ratio in chylomicrons and extremely large VLDL | 0.53 | 0.30 - 0.94 | 0.84 | 0.61 - 1.15 | 0.93 | 0.68 - 1.26 | 0.83 | 0.67 - 1.02 | 0.06963 |
| Lipoprotein subclasses | Triglycerides in chylomicrons and extremely large VLDL | 0.74 | 0.54 - 1.02 | 0.71 | 0.56 - 0.91 | 0.91 | 0.74 - 1.11 | 0.81 | 0.70 - 0.93 | 0.00268 |

**Table S3. Serum/plasma metabolites associated with VTDR**

| Category of metabolites | Name of metabolytes | Chinese | | Malay | | Indian | | Pooled | | | After Bonferroni Correction |
|---|---|---|---|---|---|---|---|---|---|---|---|
| | | OR | 95%CI | OR | 95%CI | OR | 95%CI | OR | 95%CI | P - value | |
| Apolipoproteins | Apolipoprotein A-I | 0.55 | 0.34 - 0.88 | 0.82 | 0.61 - 1.11 | 0.97 | 0.76 - 1.23 | 0.85 | 0.71 - 1.01 | 0.06079 | |
| Ketone bodies | 3-hydroxybutyrate | 2.76 | 1.67 - 4.56 | 1.29 | 0.97 - 1.71 | 1.16 | 0.90 - 1.49 | 1.34 | 1.13 - 1.60 | 0.00097 | select |
| Fluid Balance | Creatinine | 1.79 | 1.08 - 2.97 | 1.79 | 1.30 - 2.47 | 2.14 | 1.65 - 2.76 | 1.96 | 1.63 - 2.37 | 0.00000 | select |
| Glycolysis related metabolites | Glucose | 1.16 | 0.68 - 1.97 | 1.85 | 1.21 - 2.83 | 1.07 | 0.83 - 1.39 | 1.23 | 1.00 - 1.51 | 0.04508 | |
| Cholesterol | Total cholesterol in HDL2 | 0.59 | 0.35 - 1.00 | 0.74 | 0.55 - 1.00 | 0.90 | 0.70 - 1.15 | 0.80 | 0.67 - 0.95 | 0.01367 | |
| Cholesterol | Total cholesterol in HDL | 0.60 | 0.38 - 0.93 | 0.77 | 0.57 - 1.04 | 0.93 | 0.73 - 1.18 | 0.82 | 0.69 - 0.97 | 0.02154 | |
| Lipoprotein subclasses | Total cholesterol to total lipids ratio in IDL | 0.73 | 0.49 - 1.09 | 0.69 | 0.50 - 0.96 | 0.89 | 0.70 - 1.12 | 0.80 | 0.67 - 0.95 | 0.01045 | |
| Lipoprotein subclasses | Cholesterol esters to total lipids ratio in IDL | 0.71 | 0.46 - 1.09 | 0.66 | 0.48 - 0.90 | 0.84 | 0.66 - 1.06 | 0.76 | 0.64 - 0.90 | 0.00157 | select |
| Glycerides and phospholipids & Lipoprotein subclasses | Triglycerides in IDL | 1.11 | 0.71 - 1.74 | 1.53 | 1.15 - 2.04 | 1.32 | 1.04 - 1.68 | 1.36 | 1.14 - 1.61 | 0.00044 | select |
| Lipoprotein subclasses | Triglycerides in large LDL | 1.02 | 0.65 - 1.60 | 1.47 | 1.10 - 1.95 | 1.30 | 1.00 - 1.68 | 1.31 | 1.10 - 1.56 | 0.00281 | |
| Lipoprotein subclasses | Triglycerides to total lipids ratio in large LDL | 1.31 | 0.84 - 2.04 | 1.41 | 1.05 - 1.90 | 1.16 | 0.92 - 1.48 | 1.26 | 1.06 - 1.50 | 0.00744 | |
| Lipoprotein subclasses | Cholesterol esters to total lipids ratio in chylomicrons and large VLDL | 1.21 | 0.74 - 1.97 | 1.69 | 1.23 - 2.32 | 1.11 | 0.88 - 1.39 | 1.27 | 1.07 - 1.51 | 0.00682 | |

| | | | | | | | | | | | |
|---|---|---|---|---|---|---|---|---|---|---|---|
| Lipoprotein subclasses | Total cholesterol in medium HDL | 0.66 | 0.43 - 1.03 | 0.63 | 0.47 - 0.85 | 0.95 | 0.75 - 1.22 | 0.78 | 0.66 - 0.93 | 0.00624 | |
| Lipoprotein subclasses | Total cholesterol to total lipids ratio in medium HDL | 0.84 | 0.55 - 1.29 | 0.68 | 0.50 - 0.92 | 0.88 | 0.69 - 1.14 | 0.80 | 0.67 - 0.96 | 0.01373 | |
| Lipoprotein subclasses | Cholesterol esters in medium HDL | 0.64 | 0.41 - 1.01 | 0.62 | 0.46 - 0.83 | 0.94 | 0.74 - 1.21 | 0.77 | 0.64 - 0.91 | 0.00303 | |
| Lipoprotein subclasses | Cholesterol esters to total lipids ratio in medium HDL | 0.86 | 0.56 - 1.33 | 0.66 | 0.49 - 0.89 | 0.84 | 0.65 - 1.09 | 0.78 | 0.65 - 0.93 | 0.00585 | |
| Lipoprotein subclasses | Free cholesterol in medium HDL | 0.68 | 0.42 - 1.09 | 0.69 | 0.51 - 0.93 | 0.97 | 0.76 - 1.25 | 0.82 | 0.69 - 0.98 | 0.02895 | |
| Lipoprotein subclasses | Total lipids in medium HDL | 0.61 | 0.38 - 0.97 | 0.63 | 0.46 - 0.85 | 1.00 | 0.79 - 1.26 | 0.80 | 0.67 - 0.95 | 0.01233 | |
| Lipoprotein subclasses | Triglycerides to total lipids ratio in medium HDL | 1.04 | 0.68 - 1.60 | 1.41 | 1.07 - 1.86 | 1.14 | 0.90 - 1.44 | 1.21 | 1.03 - 1.43 | 0.02253 | |
| Lipoprotein subclasses | Triglycerides in medium LDL | 1.02 | 0.65 - 1.62 | 1.38 | 1.02 - 1.88 | 1.29 | 1.01 - 1.66 | 1.28 | 1.07 - 1.53 | 0.00738 | |
| Lipoprotein subclasses | Triglycerides to total lipids ratio in medium LDL | 1.28 | 0.84 - 1.97 | 1.34 | 1.00 - 1.79 | 1.15 | 0.92 - 1.45 | 1.23 | 1.04 - 1.45 | 0.01336 | |
| Lipoprotein subclasses | Phospholipids to total lipids ratio in chylomicrons and medium VLDL | 1.16 | 0.77 - 1.74 | 1.37 | 1.04 - 1.81 | 1.17 | 0.89 - 1.54 | 1.24 | 1.04 - 1.48 | 0.01485 | |
| Fatty acids by saturation (%) | Ratio of saturated fatty acids to total fatty acids | 0.70 | 0.42 - 1.16 | 0.73 | 0.54 - 0.98 | 0.71 | 0.56 - 0.90 | 0.71 | 0.60 - 0.85 | 0.00015 | select |
| Lipoprotein subclasses | Free cholesterol in small HDL | 0.69 | 0.44 - 1.09 | 0.70 | 0.53 - 0.93 | 0.95 | 0.74 - 1.21 | 0.81 | 0.68 - 0.96 | 0.01749 | |
| Lipoprotein subclasses | Phospholipids in small HDL | 0.71 | 0.44 - 1.13 | 0.66 | 0.50 - 0.89 | 0.97 | 0.76 - 1.23 | 0.81 | 0.68 - 0.96 | 0.01808 | |

| | | | | | | | | | | | |
|---|---|---|---|---|---|---|---|---|---|---|---|
| Aromatic amino acids | Tyrosine | 0.54 | 0.34 - 0.85 | 0.74 | 0.55 - 0.99 | 0.72 | 0.58 - 0.90 | 0.70 | 0.59 - 0.83 | 0.00002 | select |
| Lipoprotein particle size | Mean diameter for VLDL particles | 0.90 | 0.59 - 1.37 | 0.72 | 0.54 - 0.97 | 0.92 | 0.73 - 1.16 | 0.85 | 0.72 - 1.00 | 0.05348 | |
| Lipoprotein subclasses | Triglycerides in very small VLDL | 1.06 | 0.69 - 1.63 | 1.39 | 1.05 - 1.85 | 1.22 | 0.97 - 1.55 | 1.25 | 1.06 - 1.48 | 0.00852 | |
| Lipoprotein subclasses | Total cholesterol to total lipids ratio in chylomicrons and extremely large VLDL | 1.41 | 0.80 - 2.48 | 1.74 | 1.19 - 2.53 | 1.35 | 1.06 - 1.72 | 1.45 | 1.20 - 1.76 | 0.00015 | select |
| Lipoprotein subclasses | Cholesterol esters to total lipids ratio in chylomicrons and extremely large VLDL | 1.32 | 0.78 - 2.21 | 1.86 | 1.25 - 2.75 | 1.36 | 1.05 - 1.77 | 1.47 | 1.20 - 1.79 | 0.00016 | select |
| Lipoprotein subclasses | Triglycerides to total lipids ratio in chylomicrons and extremely large VLDL | 0.69 | 0.30 - 1.59 | 0.58 | 0.41 - 0.82 | 0.74 | 0.57 - 0.97 | 0.68 | 0.55 - 0.83 | 0.00019 | select |

**Table S4. Urinary metabolites associated with any DR**

| Category of metabolites | Name of metabolytes | Chinese | | Indian | | Pooled | | | After Bonferroni Correction |
|---|---|---|---|---|---|---|---|---|---|
| | | OR | 95%CI | OR | 95%CI | OR | 95%CI | P - value | |
| Glycolysis related metabolites | Citrate | 1.28 | 1.01 - 1.63 | 1.33 | 1.08 - 1.64 | 1.31 | 1.12 - 1.53 | 0.00073 | select |
| Miscellaneous | Ethanolamine | 1.35 | 1.04 - 1.77 | 1.28 | 1.09 - 1.49 | 1.30 | 1.13 - 1.48 | 0.00015 | select |
| Miscellaneous | Formate | 1.53 | 1.08 - 2.18 | 1.30 | 1.09 - 1.54 | 1.34 | 1.15 - 1.57 | 0.00023 | select |
| Other amino acids | Glutamine | 1.33 | 1.01 - 1.74 | 1.16 | 1.00 - 1.36 | 1.20 | 1.05 - 1.38 | 0.00702 | select |
| Miscellaneous | Hypoxanthine | 1.34 | 1.02 - 1.74 | 1.25 | 1.06 - 1.47 | 1.27 | 1.11 - 1.46 | 0.00065 | select |
| Other amino acids | Taurine | 1.15 | 0.85 - 1.56 | 1.21 | 1.01 - 1.44 | 1.19 | 1.02 - 1.39 | 0.02418 | |
| Microbial metabolism | Trimethylamine-N-oxide | 0.84 | 0.64 - 1.11 | 0.85 | 0.73 - 0.99 | 0.85 | 0.74 - 0.97 | 0.01390 | |

**Table S5. Urinary metabolites associated with moderate and above DR**

| Category of metabolites | Name of metabolytes | Chinese | | Indian | | Pooled | | | After Bonferroni Correction |
|---|---|---|---|---|---|---|---|---|---|
| | | OR | 95%CI | OR | 95%CI | OR | 95%CI | P - value | |
| Other amino acids | Alanine | 1.32 | 0.90 - 1.93 | 1.53 | 1.22 - 1.92 | 1.47 | 1.21 - 1.79 | 0.00010 | select |
| Pyrimidine metabolism | 3-Hydroxyisobutyrate | 1.29 | 0.91 - 1.82 | 1.55 | 1.25 - 1.92 | 1.48 | 1.23 - 1.77 | 0.00003 | select |
| Miscellaneous | 3-Hydroxyisovalerate | 1.39 | 0.86 - 2.22 | 1.46 | 1.22 - 1.76 | 1.45 | 1.23 - 1.73 | 0.00002 | select |
| Glycolysis related metabolites | Citrate | 1.17 | 0.84 - 1.62 | 1.99 | 1.50 - 2.63 | 1.59 | 1.29 - 1.97 | 0.00002 | select |
| Miscellaneous | Ethanolamine | 1.46 | 1.00 - 2.12 | 1.95 | 1.52 - 2.49 | 1.78 | 1.45 - 2.19 | 0.00000 | select |
| Miscellaneous | Formate | 1.56 | 0.97 - 2.51 | 1.84 | 1.43 - 2.36 | 1.78 | 1.43 - 2.21 | 0.00000 | select |
| Other amino acids | Glutamine | 1.22 | 0.85 - 1.77 | 1.31 | 1.02 - 1.67 | 1.28 | 1.05 - 1.57 | 0.01718 | |
| Miscellaneous | Glycolic acid | 1.07 | 0.75 - 1.53 | 1.44 | 1.15 - 1.79 | 1.33 | 1.10 - 1.60 | 0.00333 | select |
| Miscellaneous | Hypoxanthine | 1.43 | 0.99 - 2.07 | 1.67 | 1.28 - 2.18 | 1.59 | 1.28 - 1.97 | 0.00003 | select |
| Branched-chain amino acids | Leucine | 1.15 | 0.79 - 1.69 | 1.32 | 1.04 - 1.67 | 1.27 | 1.04 - 1.55 | 0.01915 | |
| Pyrimidine metabolism | Uracil | 1.08 | 0.74 - 1.57 | 1.36 | 1.08 - 1.72 | 1.28 | 1.05 - 1.56 | 0.01584 | |
| Branched-chain amino acids | Valine | 1.20 | 0.84 - 1.7 | 1.28 | 1.04 - 1.58 | 1.26 | 1.05 - 1.51 | 0.01261 | |

**Table S6. Urinary metabolites associated with VTDR**

| Category of metabolites | Name of metabolytes | Chinese | | Indian | | Pooled | | | After Bonferroni Correction |
|---|---|---|---|---|---|---|---|---|---|
| | | OR | 95%CI | OR | 95%CI | OR | 95%CI | P - value | |
| Other amino acids | Alanine | 1.01 | 0.60 - 1.68 | 1.41 | 1.10 - 1.81 | 1.32 | 1.06 - 1.66 | 0.01433 | |
| Miscellaneous | 3-Hydroxyisobutyrate | 1.13 | 0.70 - 1.82 | 1.44 | 1.14 - 1.82 | 1.38 | 1.11 - 1.70 | 0.00295 | select |
| Miscellaneous | 3-Hydroxyisovalerate | 1.39 | 0.76 - 2.55 | 1.48 | 1.22 - 1.80 | 1.47 | 1.22 - 1.77 | 0.00004 | select |
| Glycolysis related metabolites | Citrate | 1.05 | 0.65 - 1.69 | 1.91 | 1.41 - 2.57 | 1.61 | 1.25 - 2.08 | 0.00023 | select |
| Miscellaneous | Ethanolamine | 1.59 | 0.96 - 2.65 | 1.80 | 1.37 - 2.36 | 1.75 | 1.38 - 2.22 | 0.00001 | select |
| Miscellaneous | Formate | 1.17 | 0.61 - 2.22 | 1.81 | 1.38 - 2.37 | 1.70 | 1.32 - 2.18 | 0.00003 | select |
| Miscellaneous | Hypoxanthine | 1.37 | 0.85 - 2.21 | 2.00 | 1.45 - 2.75 | 1.78 | 1.37 - 2.32 | 0.00002 | select |
| Branched-chain amino acids | Leucine | 1.13 | 0.68 - 1.87 | 1.33 | 1.02 - 1.73 | 1.28 | 1.01 - 1.62 | 0.03892 | |
| Dietary metabolites | 3-Methylhistidine | 0.65 | 0.44 - 0.96 | 0.88 | 0.70 - 1.11 | 0.81 | 0.66 - 0.99 | 0.04364 | |
| Pyrimidine metabolism | Uracil | 1.05 | 0.63 - 1.74 | 1.54 | 1.17 - 2.02 | 1.41 | 1.11 - 1.79 | 0.00463 | select |

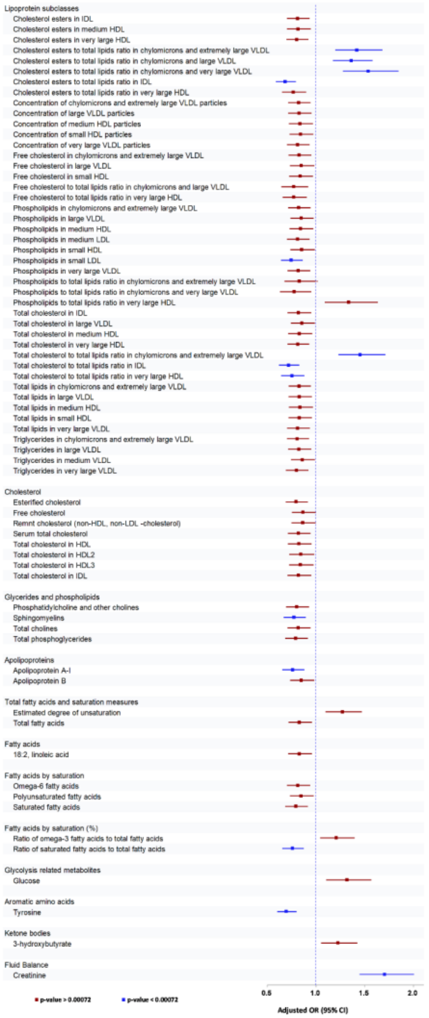

**Figure S1.** Association between serum/plasma metabolites and moderate/above diabetic retinopathy in Chinese, Indians and Malay cohorts.

OR estimate corresponds to per SD ↑ in serum metabolites
OR estimates adjusted for age, sex, systolic BP, duration of diabetes and HbA1c%

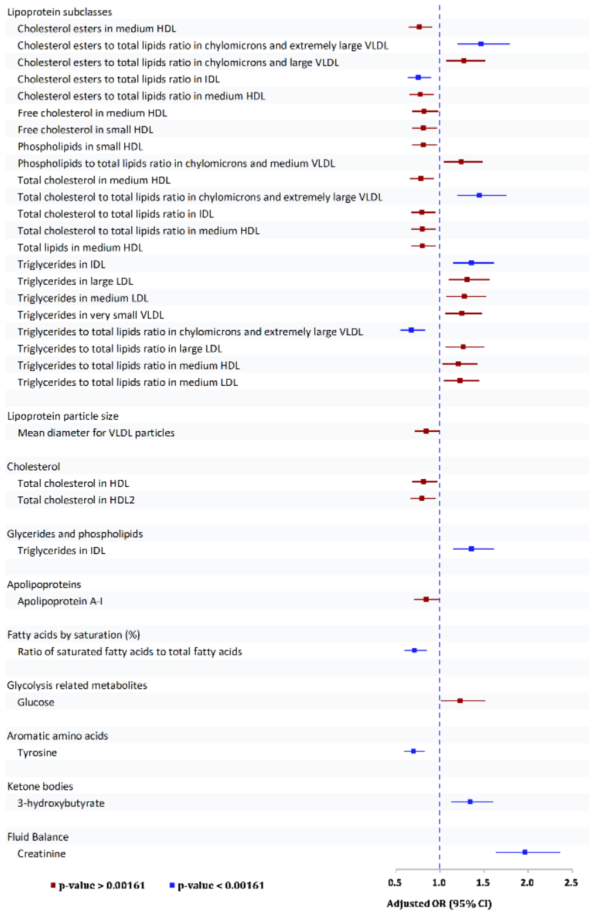


**Figure S2.** Association between serum/plasma metabolites and vision-threatening diabetic retinopathy in Chinese, Indians and Malay cohorts.

OR estimate corresponds to per SD ↑ in serum metabolites, and per SD ↓ in urine metabolites OR estimates adjusted for age, sex, systolic BP, duration of diabetes and HbA1c%
.

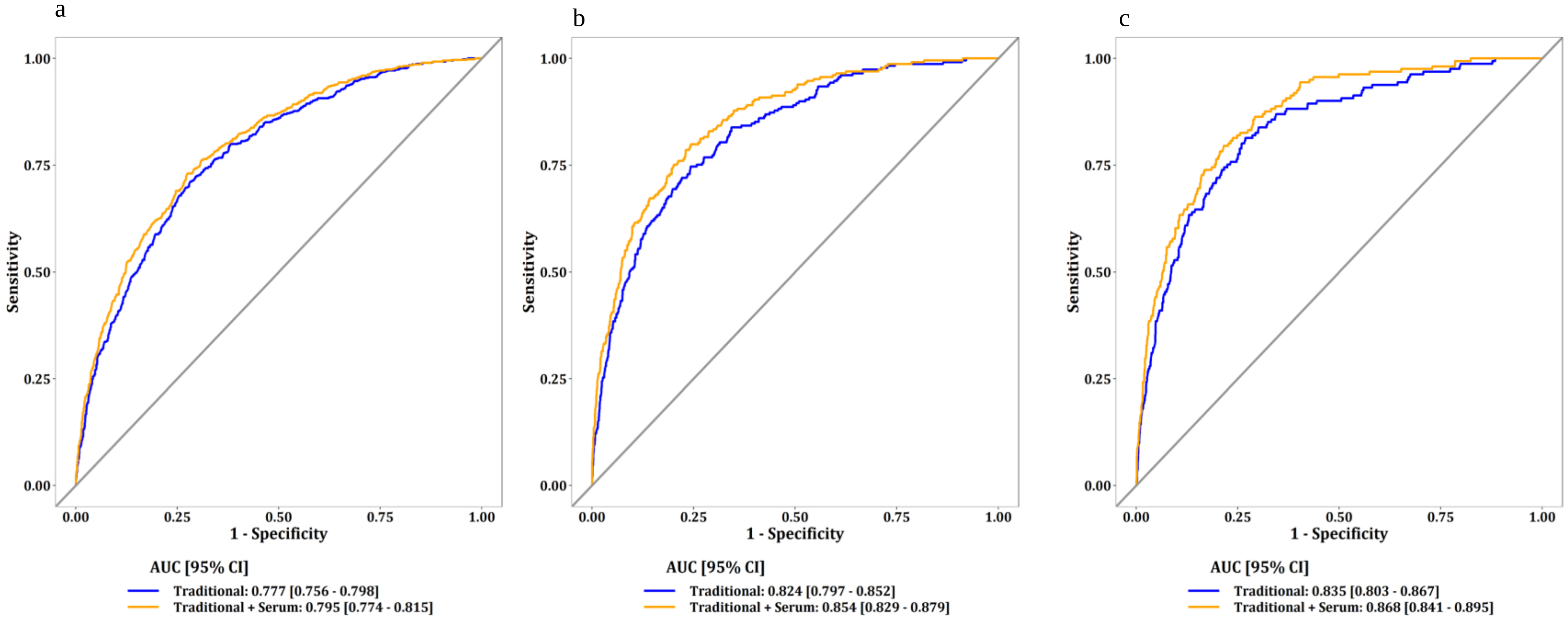


**Figure S3.** ROC curves – metabolites and a) any DR b) Moderate and above DR c) VTDR.
